# Association of Infection-Related Hospitalization With Cognitive Impairment Among Nursing Home Residents

**DOI:** 10.1001/jamanetworkopen.2021.7528

**Published:** 2021-04-23

**Authors:** Tadeja Gracner, Mansi Agarwal, Komal P. Murali, Patricia W. Stone, Elaine L. Larson, E. Yoko Furuya, Jordan M. Harrison, Andrew W. Dick

**Affiliations:** 1 RAND Corporation, Arlington, Virginia; 2 Now with RAND Corporation, Santa Monica, California; 3 Center for Health Policy, Columbia University School of Nursing, New York, New York; 4 Now with Washington University School of Medicine, St Louis, Missouri; 5 Columbia University School of Nursing, New York, New York; 6 Columbia University Mailman School of Public Health, New York, New York; 7 Department of Medicine, Columbia University Irving Medical Center, New York, New York; 8 RAND Corporation, Pittsburgh, Pennsylvania; 9 RAND Corporation, Boston, Massachusetts

## Abstract

**Importance:**

Hospitalizations for infections among nursing home (NH) residents remain common despite national initiatives to reduce them. Cognitive impairment, which markedly affects quality of life and caregiving needs, has been associated with hospitalizations, but the association between infection-related hospitalizations and long-term cognitive function among NH residents is unknown.

**Objective:**

To examine whether there are changes in cognitive function before vs after infection-related hospitalizations among NH residents.

**Design, Setting, and Participants:**

This cohort study used data from the Minimum Data Set 3.0 linked to Medicare hospitalization data from 2011 to 2017 for US nursing home residents aged 65 years or older who had experienced an infection-related hospitalization and had at least 2 quarterly Minimum Data Set assessments before and 4 or more after the infection-related hospitalization. Analyses were performed from September 1, 2019, to December 21, 2020.

**Exposure:**

Infection-related hospitalization lasting 1 to 14 days.

**Main Outcomes and Measures:**

Using an event study approach, associations between infection-related hospitalizations and quarterly changes in cognitive function among NH residents were examined overall and by sex, age, Alzheimer disease and related dementias (ADRD) diagnosis, and sepsis vs other infection-related diagnoses. Resident-level cognitive function was measured using the Cognitive Function Scale (CFS), with scores ranging from 1 (intact) to 4 (severe cognitive impairment).

**Results:**

Of the sample of 20 698 NH residents, 71.0% were women and 82.6% were non-Hispanic White individuals; the mean (SD) age at the time of transfer to the hospital was 82 (8.5) years. The mean CFS score was 2.17, and the prevalence of severe cognitive impairment (CFS score, 4) was 9.0%. During the first quarter after an infection-related hospitalization, residents experienced a mean increase of 0.06 points in CFS score (95% CI, 0.05-0.07 points; *P* < .001), or 3%. The increase in scores was greatest among residents aged 85 years or older vs younger residents by approximately 0.022 CFS points (95% CI, 0.004-0.040 points; *P* < .05). The prevalence of severe cognitive impairment increased by 1.6 percentage points (95% CI, 1.2-2.0 percentage points; *P* < .001), or 18%; the increases were observed among individuals with ADRD but not among those without it. After an infection-related hospitalization, cognition among residents who had experienced sepsis declined more than for residents who had not by about 0.02 CFS points (95% CI, 0.00-0.04 points; *P* < .05). All observed differences persisted without an accelerated rate of decline for at least 6 quarters after infection-related hospitalization. No differences were observed by sex.

**Conclusions and Relevance:**

In this cohort study, infection-related hospitalization was associated with immediate and persistent cognitive decline among nursing home residents, with the largest increase in CFS scores among older residents, those with ADRD, and those who had experienced sepsis. Identification of NH residents at risk of worsened cognition after an infection-related hospitalization may help to ensure that their care needs are addressed to prevent further cognitive decline.

## Key Points

**Question:**

Are infection-related hospitalizations associated with sustained changes in cognitive function among nursing home residents?

**Findings:**

In this cohort study of administrative data for 20 698 US nursing home residents, infection-related hospitalization was significantly associated with a sustained decrease in cognitive function, especially among those who had experienced sepsis, those aged 85 years or older, and those with Alzheimer disease and related dementias.

**Meaning:**

The findings suggest that identification of nursing home residents at highest risk of cognitive decline after an infection-related hospitalization is important to ensure that their care needs are addressed to prevent further cognitive decline.

## Teaser

This cohort study uses data from the Minimum Data Set and Medicare hospitalization records to assess the association between infection-related hospitalization and cognitive function among nursing home residents 65 years or older.

## Introduction

One in 4 long-term nursing home (NH) residents in the US is hospitalized annually, often owing to infections and related conditions, including sepsis, pneumonia, or urinary tract infection.^1,2^ Many transfers to the hospital are avoidable through effectively managed preventive care and infection management in the NHs. Recent national initiatives have aimed to reduce NH-associated infections and infection-related hospitalizations,^1,2,3,4,5^ which are associated with a significant burden of cognitive impairment, delirium, long-term disability, and death.^6,7,8,9,10,11,12^

Several observational studies have reported associations between cognitive decline and infection-related hospitalizations among older adults.^6,13,14,15,16,17^ However, without frequent assessments of cognitive outcomes before and after hospital discharge, assessing the direction of this association has been challenging because residents with cognitive impairment are more likely to have an infection-related hospitalization compared with patients who are cognitively intact.^18,19,20,21^ This especially holds true for older adults with cardiovascular events^22,23^ or with Alzheimer disease and related dementias (ADRD), for whom the association between hospitalization and cognitive decline is the strongest.^24,25,26^ Two studies^14,15^ have shown cognitive decline after hospitalization after adjusting for illness severity and prehospitalization cognitive function; however, the study participants were community dwelling and not NH residents, who have high rates of comorbidities and a disproportionately high risk of infection.^27,28^ Evidence regarding how cognitive function in NH residents changes after infection-related hospitalization, and whether that change is temporary, remains static, or accelerates over time, is limited.^6^ Our objectives were to examine whether infection-related hospitalization is associated with worse cognitive function in NH residents both immediately and over time and whether this association varies by sex, age, ADRD diagnosis, and infection-related diagnosis (sepsis vs other) at the time of the infection-related hospitalization.

## Methods

### Study Sample and Assignment of Exposure

In this cohort study, the study population included NH residents older than 64 years who had 1 infection-related hospitalization between January 2011 and December 2017. Participants were identified using the Minimum Data Set 3.0 (MDS), a standardized, comprehensive assessment tool used for all NH residents at admission and at routine intervals in federally licensed nursing homes in the US. The study sample included routine MDS assessments required at admission (or readmission) to the NH and quarterly thereafter. The MDS data included information on demographic characteristics, clinical status (including cognitive status), and hospitalization dates. The institutional review boards at Columbia University and the RAND Corporation approved this study and waived the need for informed consent because the use of facility-level data was deemed not human subjects research. The study followed the Strengthening the Reporting of Observational Studies in Epidemiology (STROBE) reporting guideline.

Resident MDS assessments were merged with the Chronic Conditions Segment of the Medicare Master Beneficiary Summary (MBSF-CC) file and the Medicare Provider Analysis and Review (MedPAR) file. The MBSF-CC file includes dates of the first diagnoses of chronic conditions (eg, ADRD, congestive heart failure, chronic obstructive pulmonary disease, and diabetes, cancer, stroke, or transient ischemic attack) among Medicare beneficiaries.

The MedPAR file contains claims for all Medicare Part A inpatient stays. Hospitalization dates reported in the MDS 3.0 were linked to the corresponding inpatient resident’s records in MedPAR with an 83% match rate, comparable to the rate found in another study.^29^ MedPAR also includes *International Classification of Diseases, Ninth Revision* and *International Statistical Classification of Diseases and Related Health Problems, Tenth Revision* codes used to identify infection-related hospitalizations. Hospital transfers were classified as infection-related hospitalizations if (1) the infection was the primary diagnosis and present at admission or (2) the infection was indicated as the MedPAR admitting diagnosis code and present at admission. We focused on hospital transfers for bacterial or suspected bacterial infections, which accounted for the majority of infection-related hospitalizations. In addition to all infection-related hospitalizations, we focused on the subset for which the primary diagnosis or admitting diagnosis was sepsis present at admission (eTables 7-10 in the Supplement). Hospital-acquired infections were excluded.

Nursing home residents were defined as exposed if they were aged 65 years or older, had 1 acute infection-related hospitalization (length of stay <15 days), were observed for at least 2 quarters before the infection-related hospitalization, and were discharged back to the NH after the hospitalization and lived there for at least a year. Alternative samples were used to address selection concerns by expanding the main sample to include residents who died soon after the infection-related hospitalization or by limiting the main sample to individuals who were observed for at least 6 quarters before and after the infection-related hospitalization.

### Outcomes

Using the Cognitive Function Scale (CFS), we measured cognitive performance by the following scores: 1, intact; 2, mild impairment; 3, moderate impairment; or 4, severe impairment, with an indicator variable for severe impairment (ie, CFS score of 4). We calculated the score using a performance-based cognitive screener for NH residents^30^: a Brief Interview for Mental Status score, or for individuals who could not complete the Brief Interview for Mental Status, an observer-based Cognitive Performance Scale score; both screening tools are available in the MDS 3.0.^31,32^ Details are provided in the eMethods in the Supplement.

### Confounders

Using the MDS, we created a categorical variable for age in 5-year increments (65-69, 70-74, 75-79, 80-84, 85-89, 90-94, 95-99, and ≥100) and indicators for whether the resident in the past week had an active diagnosis of hip fracture (MDS 3.0 item I3900) or hypertension (item I0700), was obese (body mass index ≥30, calculated as weight in kilograms divided by height in meters squared), or experienced shortness of breath when sitting or lying down or with exertion (item j1100a-c). Using the MBSF-CC, we created an indicator for whether by the current assessment the NH resident received a diagnosis of chronic obstructive pulmonary disease, congestive heart failure, lung or colorectal cancer, stroke or transient ischemic attack, diabetes, or ADRD. The number of chronic conditions was counted as a categorical variable (0 or 1-4) if the patient had a diagnosis of transient ischemic attack and/or diabetes, hypertension, or obesity, as was the number of years with ADRD (0, 1-3, 4-6, 7-10, 11-14, or ≥15 years).

### Statistical Analysis

Data were analyzed from September 1, 2019, to December 21, 2020. First, we performed descriptive analyses for resident characteristics and outcomes using unadjusted means for continuous variables and proportions for categorical variables that were generated for the last quarter before the infection-related hospitalization. We then estimated the association between infection-related hospitalization and cognitive changes at the NH-resident level, adopting an event study approach to obtain the magnitude of the association for each quarter after the infection-related hospitalization relative to the quarter of the infection-related hospitalization (ie, event). We estimated a linear specification using ordinary least squares, modeling cognitive outcomes as a linear function of individual indicators or fixed effects (to adjust for unobserved time-invariant factors or baseline differences); calendar year and month indicators (to adjust for nationwide secular trends and seasonality); indicators for age categories in 5-year increments (65-69 years as reference); a vector of indicators for the number of chronic conditions (0 as reference), years with ADRD (0 as reference), and other health conditions (chronic obstructive pulmonary disease, congestive heart failure, lung or colorectal cancer, or shortness of breath); and time since infection-related hospitalization.

Because we were interested in the associations between changes in cognition and time since infection-related hospitalization, we modeled time in 2 ways. To visually assess the patterns of cognitive function relative to the hospitalization quarter, we first modeled time with a series of quarterly binary indicators before and after the infection-related hospitalization for each resident. These indicators identified changes in outcomes in quarterly increments relative to the cognitive function in each resident’s hospitalization quarter (the reference quarter, or *t* = 0).

To quantify the change in cognition associated with infection-related hospitalization, the timing of the infection-related hospitalization should not be associated with changes in cognition, and no preintervention trend should be observed. However, because we observed a linear prehospitalization trend in outcomes, we sought to identify the difference between the estimated outcomes and the counterfactual trends (what would have happened if the individual had not become ill enough to be hospitalized).

In the second specification, we included a linear prehospitalization trend in cognitive outcomes and quarterly indicators measuring for time after infection-related hospitalization. To identify the counterfactual trends, we projected the linear trend into the postintervention period. Therefore, the coefficients on these quarterly indicators measured the change in outcomes after the infection-related hospitalization relative to any preexisting linear trend. The identifying assumption was that, conditional on having an infection-related hospitalization and on the confounders, the timing of the infection-related hospitalization would not be associated with outcome deviations from a linear trend.

We estimated models for each subpopulation to compare outcome patterns among women (vs men), among those who had been transferred to the hospital to treat sepsis (vs another infection-related condition), and among those who at the time of infection-related hospitalization were aged older than 85 years (vs younger) or had already received a diagnosis of ADRD (vs no diagnosis). In addition, we estimated a mean effect across posttransfer quarters by replacing post–infection-related hospitalization quarter indicators with a post–infection-related hospitalization indicator variable that equaled 1 in quarters after transfer and 0 before transfer; all other covariates remained the same. We stratified the model by the subgroups mentioned and used a 2-sided *t* test to examine whether the differences were significant.

We computed 95% CIs adjusted for clustering within nursing homes. We used Stata/MP statistical software, version 16.0 (StataCorp LLC) for analyses. Two-sided *P* values were deemed statistically significant at <.05.

We examined the sensitivity of the results to alternative sample specifications, addressing a concern of nonrandom attrition, arising primarily owing to mortality. We obtained prehospitalization descriptive statistics for NH residents who died within 1 year of the infection-related hospitalization to compare with those who lived longer than 1 year (eTable 1 in the Supplement). Residents who died soon after discharge were included, and the analyses were limited to those who died within 1 year of infection-related hospitalization (eTable 4 in the Supplement). To address the concern regarding the compositional changes in residents in the sample, we re-estimated specifications using a balanced panel of residents who had data from at least 6 quarters before and after the infection-related hospitalization or who had experienced up to 2 infection-related hospitalizations (eTable 4 in the Supplement). To address the concern of a spurious association between infection-related hospitalization and cognitive decline, we randomly assigned placebo infection-related hospitalization dates for each resident (eg, 4 quarters before their actual infection-related hospitalization) (eTable 5 in the Supplement).

## Results

### Study Sample

The study sample consisted of 20 698 NH residents older than 64 years who had 1 infection-related hospitalization between January 2011 and December 2017 and 2 or more quarterly MDS assessments before and 4 or more assessments after the infection-related hospitalization (eFigure 2 in the Supplement); 71.0% were women and 82.6% were non-Hispanic White individuals, and the mean (SD) age at the time of transfer to the hospital was 82 (8.5) years (Table). A total of 73.4% of residents were diagnosed with ADRD and 9.0% with severe cognitive impairment. We observed 2009 unique infection-related hospitalization dates in 22 distinct quarters (eFigure 1 in the Supplement); 41.0% of these were for sepsis.

**Table.** Characteristics of Nursing Home Residents in the Quarter Before Infection-Related Hospital Transfer

Characteristic^**a**^	Value, mean (SD), % (N = 20 698)	Observations, No.^b^	Data source
Age, y			
60-70	11.0 (31.3)	267 950	MDS
71-80	26.6 (44.2)	267 950	MDS
81-90	43.0 (49.5)	267 950	MDS
>90	19.4 (39.5)	267 950	MDS
Female	71.0 (45.4)	267 950	MDS
Race/ethnicity			
White	82.6 (37.9)	267 412	MDS
African American	10.5 (30.7)	267 412	MDS
Asian	1.7 (12.8)	267 412	MDS
Hispanic	3.7 (18.8)	267 412	MDS
American Indian or Alaska Native	0.9 (9.5)	267 412	MDS
Pacific Islander	0.7 (8.2)	267 412	MDS
Other health conditions			
Diabetes	54.2 (49.8)	267 950	MBSF-CC
Stroke or TIA	39.9 (49.0)	267 950	MBSF-CC
Hypertension	78.9 (40.8)	267 894	MDS
Hip fracture	1.9 (13.5)	267 919	MDS
Shortness of breath	12.1 (32.6)	267 950	MDS
Lung cancer	1.2 (11.0)	267 950	MBSF-CC
Colorectal cancer	4.0 (19.6)	267 950	MBSF-CC
COPD	45.1 (49.8)	267 950	MBSF-CC
CHF	59.9 (49.0)	267 950	MBSF-CC
ADRD	73.4 (44.2)	267 950	MBSF-CC
CFS score, mean (SD)^c^	2.17 (1.0)	267 918	MDS
CFS score of 4^c^	9.0 (28.7)	267 918	MDS

Abbreviations: ADRD, Alzheimer disease and related dementias; CFS, Cognitive Function Scale; CHF, congestive heart failure; COPD, chronic obstructive pulmonary disease; MBSF-CC, Medicare Master Beneficiary Summary File, Chronic Conditions; MDS, Minimum Data Set; TIA, transient ischemic attack.

^a^ Descriptive statistics are based on a sample of nursing home residents with 1 infection-related hospitalization who were followed up for at least 2 quarters before and 4 quarters or more after the infection-related hospitalization.

^b^ Describes the total number of observations available during our study period.

^c^ Scale ranges from 1 to 4, with impairment measured as follows: 1, intact; 2, mild; 3, moderate; or 4, severe.

### Infection-Related Hospitalization and Cognitive Outcomes

**Figure 1.**
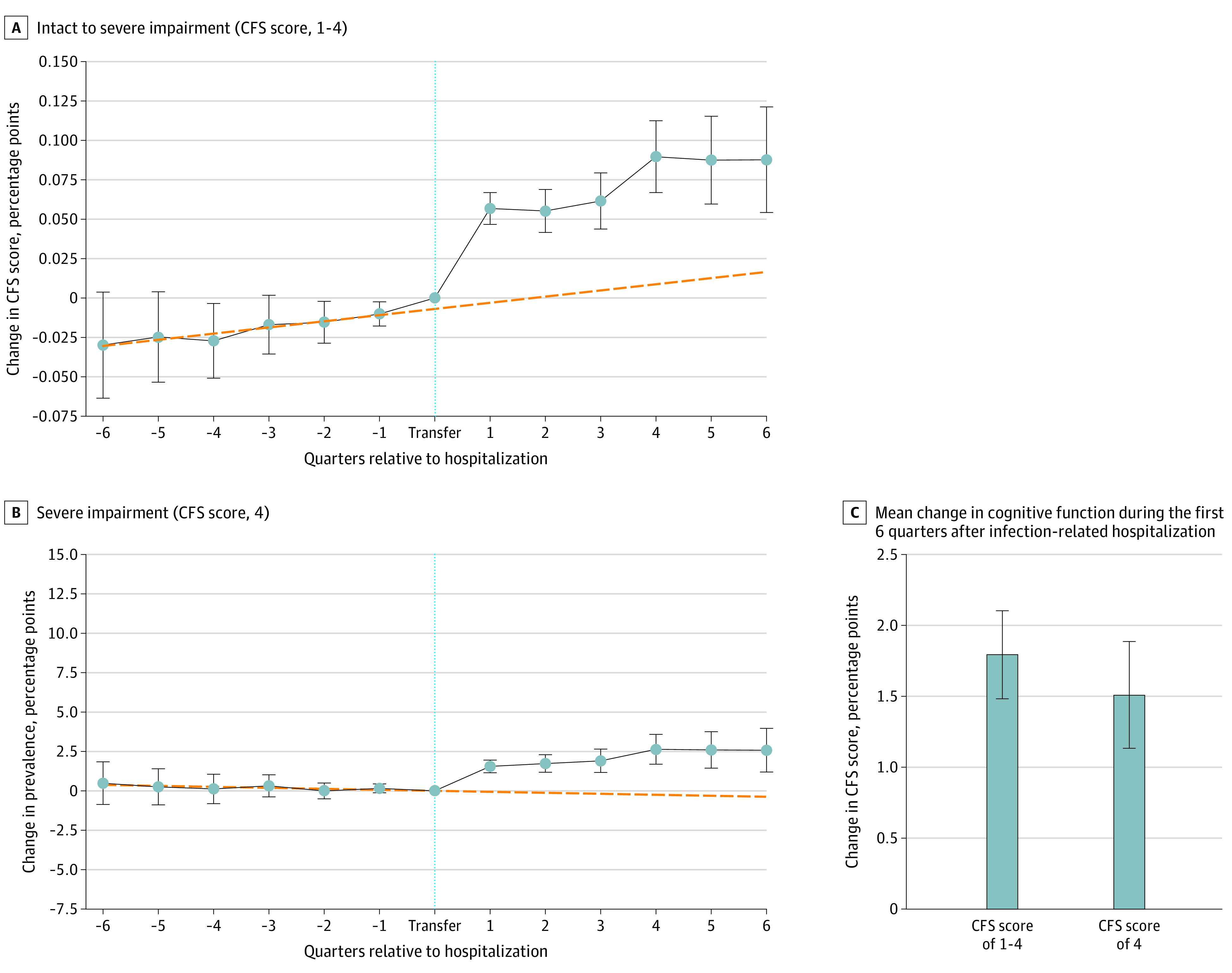
Change in Cognitive Function Before and After Infection-Related Hospitalization A and B, Markers represent the estimated effects at the event time (ie, for each quarter relative to the hospitalization quarter); whiskers, 95% CIs; dashed lines, estimated linear association between cognitive outcome and event time, indicating a counterfactual trend in the outcome in the absence of hospitalization. Regression models are provided in eTable 2 in the Supplement. C, Differences were significant at *P* < .001; the Cognitive Function Scale (CFS) score was assumed to be a cardinal metric and was normalized to vary from 0 to 1; changes in each outcome are presented as the fractional (percentage point) absolute change in the metric. Regression models are provided in eTable 3 in the Supplement.

In analysis of the association between infection-related hospitalization and cognitive outcomes, a cognitive decline of approximately 0.02 CFS points in quarters preceding the infection-related hospitalization was found (Figure 1A); no prehospitalization trend in severe cognitive impairment was observed (Figure 1C). In the first quarter after discharge to the NH, CFS scores increased by a mean of 0.06 points (95% CI, 0.05-0.07 points; *P* < .001), or 3%, and the deviation from the trend before infection-related hospitalization persisted thereafter without an increase in the rate of decline (Figure 1A and eTable 2 in the Supplement). The prevalence of severe cognitive impairment increased by 1.6 (95% CI, 1.2-2.0) percentage points—an 18% increase compared with the 9.0% prevalence before the event—in the first quarter after discharge. It then increased over time: 6 quarters after the infection-related hospitalization, the prevalence of severe cognitive impairment was higher by 2.9 percentage points (95% CI, 2.1-3.7 percentage points; *P* < .001) compared with what was expected in the absence of hospital transfer (Figure 1B). Over 6 quarters after hospitalization, CFS scores increased by a mean of 0.05 (95% CI, 0.04-0.06) points, and the prevalence of severe cognitive impairment increased by a mean of 1.5 (95% CI, 1.1-1.9) percentage points (Figure 1C).

**Figure 2.**
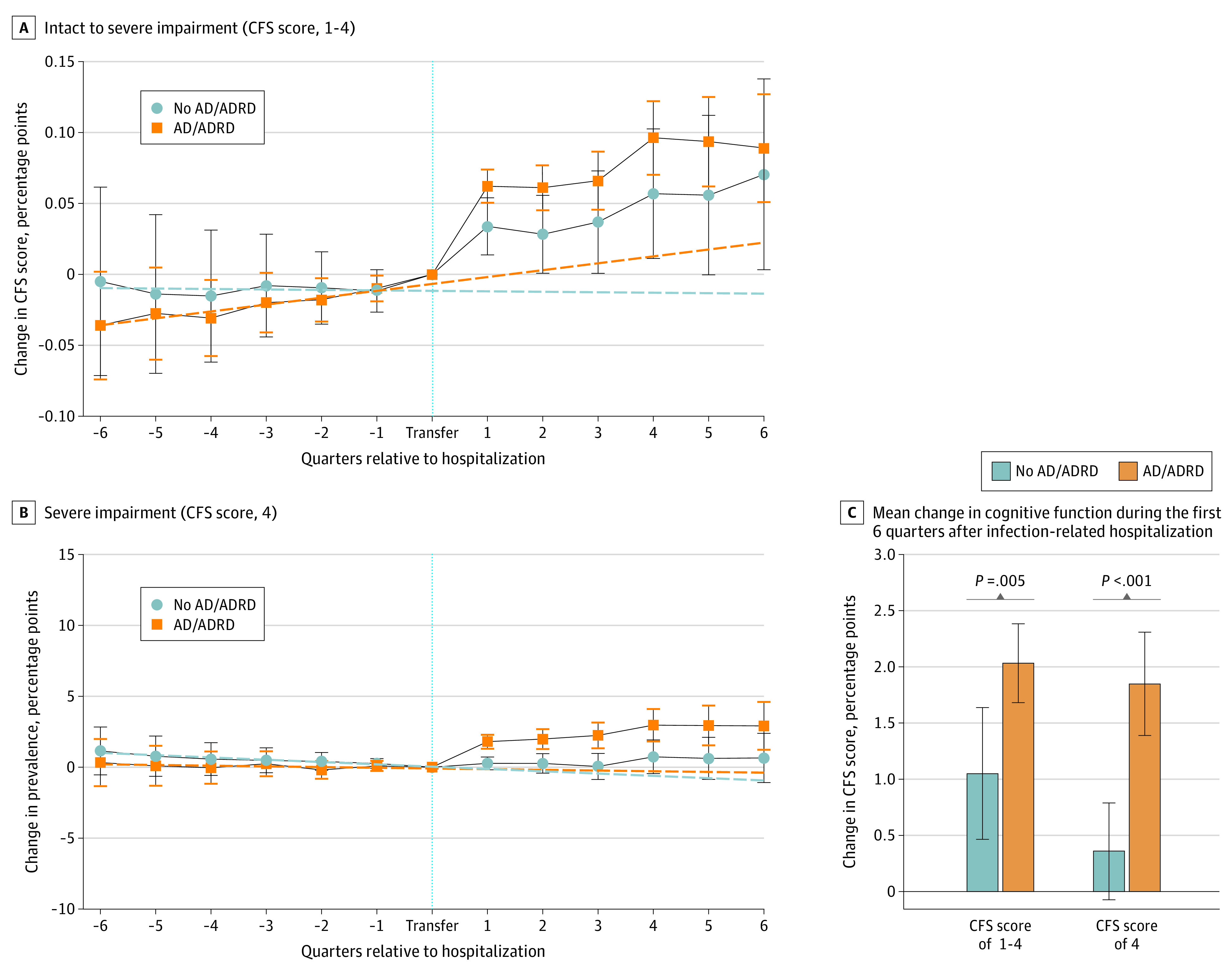
Change in Cognitive Function Before vs After Infection-Related Hospitalization by Alzheimer Disease and Related Dementia (ADRD) Status at Transfer A and B, The Cognitive Function Scale (CFS) score was assumed to be a cardinal metric and was normalized to vary from 0 to 1; changes in each outcome are presented as the fractional (percentage point) absolute change in the metric. Markers indicate effect estimates for each quarter relative to the hospitalization quarter; whiskers, 95% CIs. C, Regression models and results from tests for significant differences in coefficients between subgroups are provided in eTable 3 in the Supplement.

Although all residents experienced some level of cognitive decline after infection-related hospitalization, the decline was significantly larger for residents with diagnosed ADRD. The CFS score increased by a mean of 0.03 points (95% CI, 0.01-0.05 points; *P* = .005) more among these individuals compared with residents without ADRD during the 6 quarters after infection-related hospitalization (Figure 2A and C and eTable 3 in the Supplement). Increased severe impairment after infection-related hospitalization was associated with a diagnosis of ADRD. The prevalence of severe impairment was increased by 1.5 percentage points (95% CI, 0.9-2.1 percentage points; *P* < .001) among residents with ADRD compared with residents without ADRD, who experienced no change in severe impairment during the first 6 quarters after hospital transfer (Figure 2B and C and eTable 3 in the Supplement). Cognitive decline preceding hospitalization was observed only among residents with ADRD (Figure 2A).

Differences in cognitive function between age groups immediately after the infection-related hospitalization were observed and persisted thereafter (eFigure 3 in the Supplement). Compared with younger residents (aged 65-84 years), those 85 years or older experienced a significantly larger decline in cognitive score of 0.022 points (95% CI, 0.004-0.040 points; *P* < .05) during the 6 quarters after the infection-related hospitalization (eTable 3 in the Supplement). Differences in the prevalence of severe impairment among residents younger than 85 years vs those 85 years or older were increased 1 year after infection-related hospitalization, but these differences were not significant (eFigure 3 in the Supplement).

**Figure 3.**
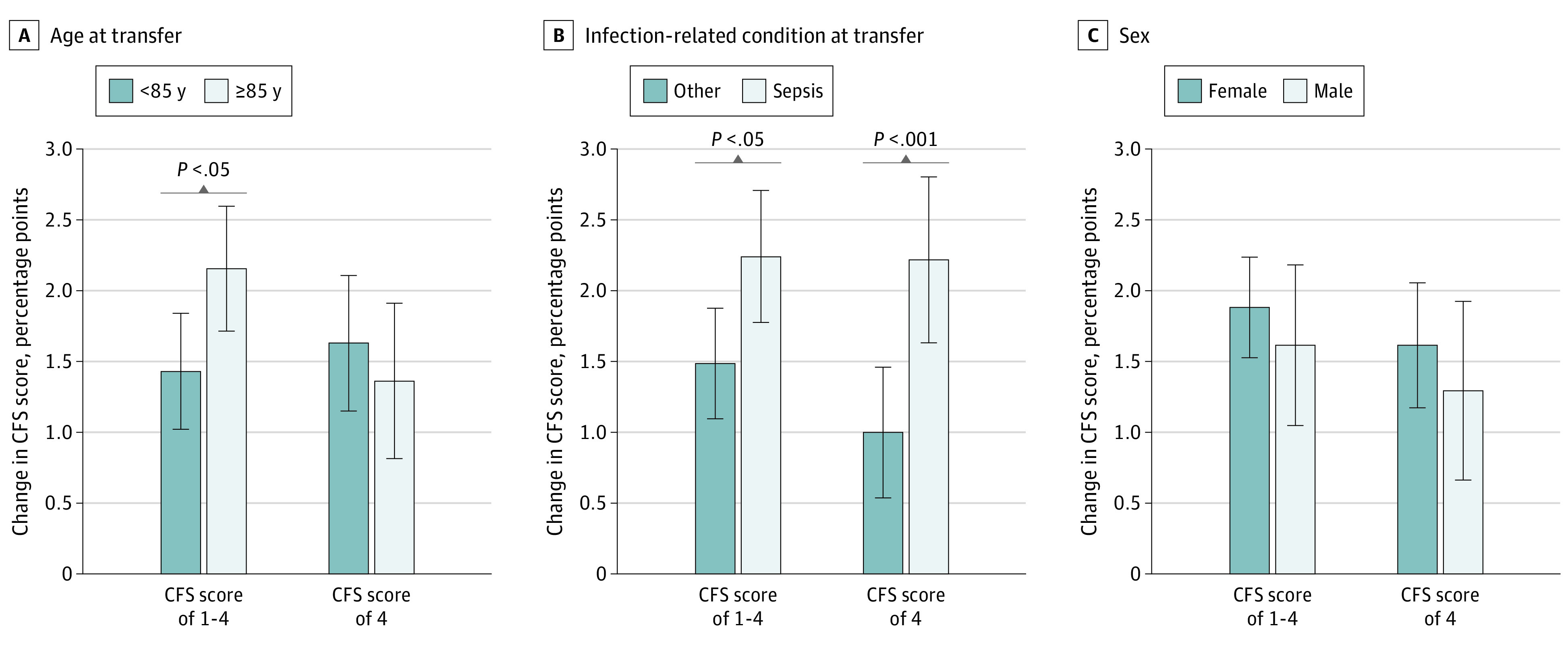
Mean Change in Cognitive Function Before vs After Infection-Related Hospitalization by Age, Sepsis, and Sex The Cognitive Function Scale (CFS) score was assumed to be a cardinal metric and was normalized to vary from 0 to 1; changes in each outcome are presented as the fractional (percentage point) absolute change in the metric. Whiskers indicate 95% CIs. Regression models and results of tests for significant differences in coefficients between subgroups are provided in eTable 4 in the Supplement.

The cognitive function of residents hospitalized for sepsis declined more than that of residents who were hospitalized for other conditions (Figure 3B and eTable 3 in the Supplement). During the 6 quarters after infection-related hospitalization, the CFS score and the prevalence of severe impairment were increased by 0.023 points (95% CI, 0.004-0.04 points; *P* < .05) and 1.2 percentage points (95% CI, 0.5-2.0 percentage points; *P* < .001) more, respectively, among those who were hospitalized with sepsis compared with those who were hospitalized for other infections. These differences observed for severe impairment were likely attributable to outcome changes during the first quarter after the infection-related hospitalization (eFigure 4 in the Supplement). No significant differences were observed by sex (Figure 3C and eTable 3 in the Supplement). Infection-related hospitalization was also associated with an increase in the prevalence of delirium (eFigure 5 in the Supplement). The mean CFS score and prevalence of severe cognitive impairment at transfer are presented by subgroups in eTable 6 in the Supplement.

### Sensitivity Analyses

When restricting the sample to residents who died within 1 year of infection-related hospitalization, the estimated changes were larger across all cognitive outcomes (eFigure 3 and eTable 4 in the Supplement), suggesting that the estimates for those who lived 1 year or longer after infection-related hospitalization may have been attenuated. The substantive findings did not change when including individuals who died within 1 year of but not earlier than 2 quarters after infection-related hospitalization, when the sample was balanced before and after infection-related hospitalization, or when allowing for up to 2 infection-related hospitalizations (eTable 4 in the Supplement). We also found no evidence of spurious correlation between cognitive outcomes and infection-related hospitalization (eTable 5 in the Supplement).

## Discussion

In this longitudinal cohort study of NH residents, infection-related hospitalization was associated with cognitive decline after the hospitalization. Within the first quarter of infection-related hospitalization, the prevalence of severe cognitive impairment was 18% higher than the prevalence expected in the absence of infection-related hospitalization. Infection-related hospitalization was associated with abrupt loss of cognitive function that persisted for up to at least 6 quarters after transfer to the hospital rather than with a steeper slope of decline over time. Cognitive decline after infection-related hospitalization was the greatest among residents who had received a diagnosis of ADRD and those 85 years or older. Individuals who were hospitalized with sepsis experienced worse cognitive outcomes immediately after hospital discharge compared with those hospitalized for other infection-related conditions.

These findings support existing evidence of an association between hospitalization and reduced cognitive function among older adults^13,14,15^ and show that cognitive function following such an event declines over time in NH residents. Compared with community-dwelling adults, NH residents are at greater risk of acquiring infections^33^ and of experiencing increased cognitive decline after infection-related hospitalization owing to higher frailty or compromised cognitive function before hospital transfer.^18^ In addition to infection,^34^ hospital-related delirium, depression, stress, polypharmacy, and isolation from caregivers may also be associated with impaired cognition. Delirium in particular is associated with accelerated cognitive decline and increased likelihood of hospitalization or rehospitalization.^17^ The risk for cognitive decline (with or without infection-related hospitalization) may be greater among elderly individuals or those with an underlying diagnosis of ADRD, who account for more than 50% of all residents in NHs^13,14,15,24,35,36^; thus, the direction of associations is less clear.

The findings of the present study suggest that careful monitoring of cognitive function before and after infection-related hospitalization, particularly for NH residents at high risk for cognitive decline, is imperative. Cross-sectional or retrospective assessments of prehospitalization cognitive function are limited because they do not address reverse-causality concerns^37^ and because family members and even health care professionals often miss early signs of cognitive decline; thus, whether infection-related hospitalization is associated with the loss of cognitive function or is simply a marker of cognitive decline that has not yet been diagnosed is unclear.^37^ Similar to studies by Ehlenbach et al^13^ and Girard et al,^14^ who examined older adults over time, the current study adjusted for prehospitalization cognitive scores and other health conditions and found an abrupt loss of cognitive function after infection-related hospitalization among NH residents who survive, suggesting that the infection-related hospitalization was associated with the loss of cognitive function.

Although these findings should not be interpreted in such a way as to diminish the necessary role of hospitalizations for appropriate treatment of infection, they lend support to the view that multidisciplinary national initiatives aimed at reducing NH-associated infections and avoidable hospitalizations such as those related to infection are imperative. Infection-related hospitalizations are largely avoidable; superior outcomes have been found through effectively managed preventive care in the NH.^1,11,38,39,40^ However, despite decreased hospitalization rates of NH residents overall,^41^ infection-related hospitalizations increased by 7% from 2011 to 2017.^42^ Strengthening programs for infection control and management within NHs thus remains a national priority,^43^ and the COVID-19 pandemic has highlighted the need. As shown during the pandemic, many NHs still lack the resources and infrastructure needed to fully support early and comprehensive infection control and treatment.^44,45,46^

### Limitations

This study has limitations. Although an immediate change in outcomes after infection-related hospitalization and the sensitivity analyses may provide confidence in our findings, unavailability of a control group (ie, comparable NH residents without an infection-related hospitalization) and the observational study design prevented us from determining causality.^11^ Mortality after hospital transfer in the NH population is high,^47,48,49,50^ and this study’s results were limited to healthier individuals who lived at least 1 year after hospitalization; therefore, the association between infection-related hospitalization and cognition was likely underestimated. Because of the inability to observe CFS scores higher than 4, detection of outcomes in individuals who were most severely impaired was difficult. We were not able to identify the exact mechanisms underlying this study’s findings. Although infection is an important factor associated with cognitive decline,^34^ hospital transfer or other mediators could also be burdensome.

## Conclusions

The findings of this study suggest that infection-related hospitalization is associated with cognitive decline among NH residents after returning to the NH. Better care structures appear to be needed to reduce infection-related hospitalization to prevent cognitive decline. Better monitoring of cognitive function and interventions targeted toward residents at greatest risk of cognitive decline may help improve NH quality of care and resident outcomes.

## Notes

Supplement. **eMethods.** **eFigure 1.** Distribution of infection-related hospital transfer dates **eFigure 2.** Descriptive characteristics of the analytical sample **eFigure 3.** Share of NH residents dying after the infection-related hospital transfer **eFigure 4.** Change in cognitive function before and after infection-related hospitalization, stratified by age and infection-related condition at transfer (sepsis vs other) **eFigure 5.** Change in presence of delirium symptoms before and after infection-related hospitalization **eTable 1.** Characteristics of those surviving at least one year after, and those dying within the first or three quarters of infection-related hospitalization **eTable 2.** Association between infection-related hospitalization and cognitive function over time **eTable 3.** Average and heterogenous effect of infection-related hospitalization on cognitive function by sex, age, infection-related condition and Alzheimer's disease and related dementia at the time of transfer **eTable 4.** Association between infection-related hospitalization and cognitive function over time, using alternate samples **eTable 5.** Association between infection-related hospitalization and cognitive function over time, using placebo hospitalization dates **eTable 6.** Mean CFS scores and severe impairment prevalence at transfer by subgroups **eTable 7.** ICD-9 infection codes **eTable 8.** ICD-10 infection codes **eTable 9.** ICD-9 codes identifying sepsis-related complications **eTable 10.** ICD-10 codes identifying sepsis-related complications **eReferences**
