# Supplementary material for: Association of Infection-Related Hospitalization With Cognitive Impairment Among Nursing Home Residents
Source: JAMA Netw Open. 2021 Apr 23;4(4):e217528. doi: 10.1001/jamanetworkopen.2021.7528 (PMC8065379; doi:10.1001/jamanetworkopen.2021.7528)
Supplement: Supplement. — eMethods. eFigure 1. Distribution of infection-related hospital transfer dates eFigure 2. Descriptive characteristics of the analytical sample eFigure 3. Share of NH residents dying after the infection-related hospital transfer eFigure 4. Change in cognitive function before and after infection-related hospitalization, stratified by age and infection-related condition at transfer (sepsis vs other) eFigure 5. Change in presence of delirium symptoms before and after infection-related hospitalization eTable 1. Characteristics of those surviving at least one year after, and those dying within the first or three quarters of infection-related hospitalization eTable 2. Association between infection-related hospitalization and cognitive function over time eTable 3. Average and heterogenous effect of infection-related hospitalization on cognitive function by sex, age, infection-related condition and Alzheimer's disease and related dementia at the time of transfer eTable 4. Association between infection-related hospitalization and cognitive function over time, using alternate samples eTable 5. Association between infection-related hospitalization and cognitive function over time, using placebo hospitalization dates eTable 6. Mean CFS scores and severe impairment prevalence at transfer by subgroups eTable 7. ICD-9 infection codes eTable 8. ICD-10 infection codes eTable 9. ICD-9 codes identifying sepsis-related complications eTable 10. ICD-10 codes identifying sepsis-related complications eReferences [file jamanetwopen-e217528-s001.pdf]

Supplementary Online Content

Gracner T, Agarwal M, Murali KP, et al. Association of infection-related hospitalization with cognitive impairment among nursing home residents. *JAMA Netw Open*. 2021;4(4):e217528. doi:10.1001/jamanetworkopen.2021.7528

eMethods
eFigure 1. Distribution of infection-related hospital transfer dates
eFigure 2. Descriptive characteristics of the analytical sample
eFigure 3. Share of NH residents dying after the infection-related hospital transfer
eFigure 4. Change in cognitive function before and after infection-related hospitalization, stratified by age and infection-related condition at transfer (sepsis vs other)
eFigure 5. Change in presence of delirium symptoms before and after infection-related hospitalization
eTable 1. Characteristics of those surviving at least one year after, and those dying within the first or three quarters of infection-related hospitalization
eTable 2. Association between infection-related hospitalization and cognitive function over time
eTable 3. Average and heterogenous effect of infection-related hospitalization on cognitive function by sex, age, infection-related condition and Alzheimer's disease and related dementia at the time of transfer
eTable 4. Association between infection-related hospitalization and cognitive function over time, using alternate samples
eTable 5. Association between infection-related hospitalization and cognitive function over time, using placebo hospitalization dates
eTable 6. Mean CFS scores and severe impairment prevalence at transfer by subgroups**.**
eTable 7. ICD-9 infection codes
eTable 8. ICD-10 infection codes
eTable 9. ICD-9 codes identifying sepsis-related complications
eTable 10. ICD-10 codes identifying sepsis-related complications
eReferences

This supplementary material has been provided by the authors to give readers additional information about their work.

© 2021 Gracner et al. *JAMA Network Open*.

eMethods

Section 1. Sample and exposure

Identification of infection-related hospitalizations among nursing home residents

The Minimum Data Set (MDS) 3.0 combined with the Medicare Provider Analysis and Review (MedPAR) data were used to identify NH residents aged 65 and over who had one 1-14 days long hospital transfer due to infection sometime between January 1st, 2011, to December 31st, 2017. Hospital transfers reported in MDS were linked to corresponding inpatient records in MedPAR. Infections were identified based on ICD-9 and ICD-10 codes in MedPAR. Infections-related conditions were classified by site, i.e., sepsis, respiratory, urinary tract infection (UTI), gastrointestinal, skin/soft tissue, IV catheter related, and other. Hospital transfers were classified as due to infection if (1) infection was the primary diagnosis and present on admission (POA) or (2) infection was indicated as the MedPAR admitting diagnosis code and POA.

We used previously categorized ICD-9 codes[10,11], online mapping tools (ICD-9/10)[a], Stata commands[b], and CDC classifications[c]. They were reviewed by an infectious disease physician on our team (also a co-author of this study) for accuracy. Our analyses focused on hospital transfers for bacterial or suspected bacterial infections, which accounted for the vast majority of infection-related hospital transfers.

17% of hospital transfers reported in MDS did not have a corresponding inpatient record in MedPAR and were therefore missing the ICD-9 or ICD-10 codes to identify whether the transfer

[a] https://icd.codes/convert/icd10-to-icd9-cm, https://icd10coded.com/convert/?code=008.66, https://icdlist.com/convert?t=icd9&c=711.0
[b] https://www.stata.com/features/overview/icd/
[c] https://www.cdc.gov/nchs/icd/icd10cm.htm

© 2021 Gracner et al. *JAMA Network Open*.

was caused by infection-related condition or another reason; those residents were excluded from our analyses. We found that missing MedPAR data was not correlated with any observable resident characteristics. Consistent with previous work[1], we found that infection-related conditions accounted for nearly half of all hospital transfers among NH residents.

Sample and exposure

Out of 159,582 NH residents with at least one infection-related transfer (InfHosp), we first limited the study sample to those were in the data at least two and up to ten consecutive quarters before and after the InfHosp, and had CFS measure available as of January 2011 (N=114,747). We then limited our sample to NH residents for whom we observed exactly one InfHosp (N= 89,341) that was short-stay (<15 days) (N = 85,301). Analyses were limited to residents with one InfHosp to reduce the concern of secular trends in the outcome variable measuring cognitive function preceding hospitalization, which could violate our identification assumption in the statistical analyses. NH residents were considered exposed to the InfHosp if they were transferred from the NH to the hospital, stayed there between 1-14 days, and were discharged back to the NH during the study period. We assigned exposure based on the date of their first and only infection-related hospital transfer, as recorded in their MDS assessment. To observe pre-hospitalization trends along with the long-term dynamics of cognitive function after the transfer, we then finally limited the sample on residents for whom we observed assessments on cognitive and other health and socio-economic measures in at least two consecutive quarters prior to at least four after the InfHosp (N=20,698). We varied our sample restrictions in our sensitivity analyses.

© 2021 Gracner et al. *JAMA Network Open*.

The major reason for such a significant drop in our sample size after applying this condition is that a large share of NH residents died soon after the hospital transfer. Out of 58,673 NH residents who died before December 2017, 62.4% died within the quarter of InfHosp, and additional 11,701 residents died within the first three quarters since InfHosp. A remaining share of NH residents were not discharged back to the NH in our data; we do not know where to. Thus, our sample represents a selected sample of relatively healthier NH residents who survive at least one year after the transfer.

Section 2. Outcome variables

The primary outcomes measuring cognitive function of NH residents were defined using the Cognitive Function Scale (CFS) score, ranging from 1 (intact), 2 (with mild impairment), 3 (with moderate impairment) to 4 (with severe impairment). We followed prior work[2] and calculated the score using the a short performance-based cognitive screener for NH residents, a Brief Interview for Mental Status score, dividing residents into three larger categories: those with intact/borderline cognition (13–15) moderate cognitive impairment (8–12), severe cognitive impairment (0–7) [3,4]. We then calculated the Cognitive Performance Scale (CPS) score, obtained using an algorithm which assigns residents a score between 0–6 based on five MDS items (i.e., daily decision making, eating self-performance, ability to make self-understood, short term memory, and whether or not the resident is comatose). CPS score ranged between 0-6, from cognitively intact to severely impaired. Some NH residents were not able to complete the BIMS, and for them, CPS score of larger or equal than 5 was assigned [5]. Finally, we combined BIMS and CPS score into a CFS score, ranging from 1-4: the highest level of impairment ("severely impaired" or CFS 4) includes NH residents who did not complete BIMS and have a CPS score of 5 or 6. NH residents that scored

© 2021 Gracner et al. *JAMA Network Open*.

between 0-7 on the BIMS or a 3-4 on the CPS were defined as with "moderate impairments" or CFS 3. NH residents with a BIMS score of 8-12 or a CPS score of 0-2 were considered to be with "mild impairments" or CFS 2. Residents who were able to complete BIMS and who scored between 13-15 were considered "cognitively intact" of CFS 1. Based on this variable, we defined an indicator variable for severe impairment (i.e., CFS equaled 4).

Section 3. Statistical model

To visually (and flexibly) assess the pattern of cognitive outcomes relative to the quarter of InfHosp, we start by estimating the following event study model:

$$(1)\ Y_{ijt} = \alpha_0 + \sum_{q=-6}^{-1} \alpha_q + \sum_{q=1}^{6} \alpha_q + X_{it}'\theta + \gamma_i + \delta_t + \epsilon_{ijt}$$

Where $Yijt$ describes the outcome of interest for NH resident *i*, living in a nursing home *j* at time *t*, denoting calendar years and quarters. The subscript q refers to event quarters, which are quarter intervals relative to the quarter prior to InfHosp (i.e., period q = -2 refers to 2 quarters before the InfHosp), and α_q are coefficients on indicators for quarters relative to the InfHosp. Thus, this model effectively compares changes in cognitive outcomes in each quarter increment before vs after InfHosp for each NH resident – that is, it estimates the differences in outcomes for leads and lags of InfHosp relative to a reference pre-hospitalization quarter (t – 1) - these relative differences are described by the coefficients α_q for each *q*. We presented these coefficients in all of our main and supplement figures, along with 95% confidence intervals, adjusted for serial correlation in the outcome at the NH level. Controls for resident's age, coexisting medical conditions (i.e., diabetes, cancer, ischemic heart disease) as well as a binary variable indicating hip fracture or shortness of breath were also included (X_{it}'). Calendar quarterly and year indicators, δ_t, were used to adjust for

nationwide secular trends and seasonality in the cognitive outcomes (see Figure 2 and eTable 2, Panel A).

Guided by the patterns obtained using the model (1), and to summarize the magnitude of estimated effects and their statistical significance, a linear pre-trend in event time was allowed and superimposed, as described in our model (2) below:

$$(2)\ Y_{ijt} = \alpha_0 + \mu q + \sum_{q=0}^{6} \alpha_q + X_{it}'\theta + \gamma_i + \delta_t + \epsilon_{ijt}$$

Model (2) allowed for a linear pre-trend in event time q (i.e., between quarter MDS assessments). We decided for the linear trend based on the Figure 2 based on model (1), which suggested that a linear trend captures secular trends well. The key coefficient of interest here was α_q, which measured the change in cognitive function following the InfHosp relative to any pre-existing linear trend (μ). In this model (2), the identifying assumption was that, conditional on controls and having an InfHosp, the timing of the InfHosp was uncorrelated with *deviations* of the outcome from a linear trend in event time. Finally, because we observed that transfer effects on outcomes were mostly immediate and persisted over time, we also estimated an average effect of all combinations of comparisons between residents across all post-transfer quarters by replacing $\sum_{q=0}^{6} \alpha_q$ in the model (2) with an indicator variable that equaled 1 in all quarters post-transfer and 0 otherwise; all other covariates remained the same (see results in eTable 3, Panel B).

Section 4. Sensitivity Analyses.

A major concern in our analyses is non-random attrition, arising primarily due to mortality. It poses a potential threat to our identifying assumption if it is correlated with our outcome of interest, in either levels or changes. This is likely, if hospitalizations for NH residents with worse pre-hospitalization cognitive outcomes are also more likely to result in death. eFigure 3 shows that a large share of NH residents indeed died within the first quarter since InfHosp. eTable 1 compares residents who died in the quarter or within the first three quarters of InfHosp and shows that though on average similar in characteristics like race and co-morbidities, residents who died in the quarter of InfHosp had worse cognitive function prior to InfHosp than did the long-term InfHosp survivors (e.g., 13.7% vs 9% with severe cognitive impairment). They were also older (84.4 vs 82.7), less likely to be female (64.1% vs 70.9%), and majority were transferred due to sepsis: 67% vs 41%. When restricting our study sample to those who died within one year of transfer, our estimates show significant changes across all cognitive outcomes (see eTable 3, panel D). For instance, residents experienced 0.11 point increase in CFS (CI95% 0.103 – 0.124, p<0.001; vs about 0.05 for those who survived past one year), their likelihood of severe impairment increased by about 4pp (CI95% 0.35 – 0.044, p<0.001; vs about 2.5pp among survivors). These results combined suggest that our estimates on survivors may be underestimating the effect of InfHosp on cognitive function.

We varied the sample restrictions to NH residents who survived up to at least 2, 6, or 8 quarters post InfHosp, and results remained similar across alternative samples; about 2% of residents die with each additional quarter. Using individual fixed approach further addressed potential bias due to non-random attrition because the impact of a hospital admission was estimated entirely off of

© 2021 Gracner et al. *JAMA Network Open*.

within-individual changes and therefore should not be contaminated by any differential attrition correlated with the level of the outcome. We further investigated the potential concern regarding the compositional changes in the set of individuals used to identify our estimates by re-estimating our specifications on a balanced panel of residents observed in at least 6 quarters prior and after the InfHosp. Estimation on this balanced panel allows us to examine the time pattern of outcomes without concerns about potential effects of compositional sample changes (eTable 4).

Finally, to address the concern of spurious correlation between InfHosp and cognitive decline, we also reproduced the analysis under conditions in which it was unlikely to demonstrate our findings. Specifically, we assigned random placebo hospitalization dates (e.g., we changed hospitalization dates to 4 quarters back from their actual date). We found no significant relationship between them and outcomes of interest. See eTable 5 for results.

Section 6. Infection-related hospitalization and delirium symptoms.

We followed the Confusion Assessment Method (CAM) criteria (validated with high sensitivity, specificity and reliability in previous work[6-9] when identifying residents with delirium. Specifically, following Kosar et al.[9], we defined delirium as present if patient was reported to have demonstrated an acute change or fluctuation in delirium symptoms, such as inattention, and either disorganized thinking or an altered level of consciousness (i.e., MDS variables c1300a/b/c/d equal to 2, indicating one or more delirium signs and symptoms that fluctuate present), or if there was evidence of an acute change in mental status from the resident's baseline (c1600 equals 1). eFigure 5A below shows that increased prevalence of delirium symptoms post-InfHosp is short-lived: we observe a significant increase of about 1 percentage point in fluctuating delirium symptoms during

© 2021 Gracner et al. *JAMA Network Open*.

the first quarter post-InfHosp that return to their pre-hospitalization trajectory after that. In contrast, following InfHosp delirium symptoms that are both, continuously present or fluctuating (defined with the MDS variables above, and if variables c1300a/b/c/d equal to 1; indicating that one or more delirium signs and symptoms are continuously present), increase permanently. Specifically, we found that even one year after InfHosp, the probability of experiencing delirium symptoms that are continuously present is higher by nearly 1.5 percentage points than it would have been in the absence of InfHosp (eFigure 5B).

© 2021 Gracner et al. *JAMA Network Open*.

eFigure 1: Distribution of infection-related hospital transfer dates.

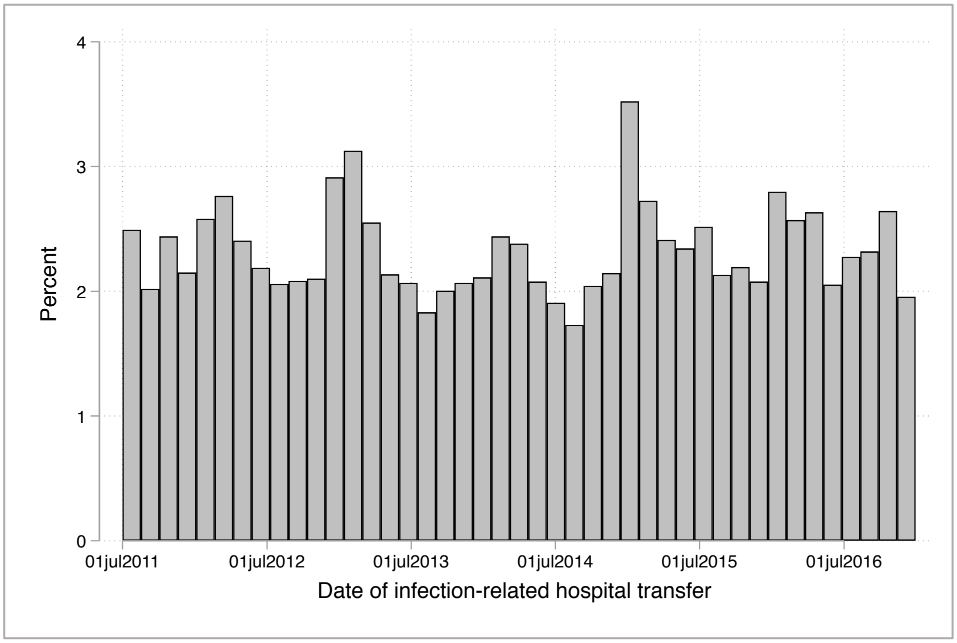

Notes: Distribution of infection-related hospital transfer dates is based on a sample of NH residents with one short-stay InfHosp, and who were followed for at least two quarters prior and four or more after the InfHosp. Hospitalizations dates in the final analytic sample thus run from July 2011 and December 2016, with resident outcomes followed from January 2011 through December 2017.

© 2021 Gracner et al. *JAMA Network Open*.

eFigure 2. Descriptive characteristics of the analytical sample.

(A) Distribution of quarters to or since hospital transfer

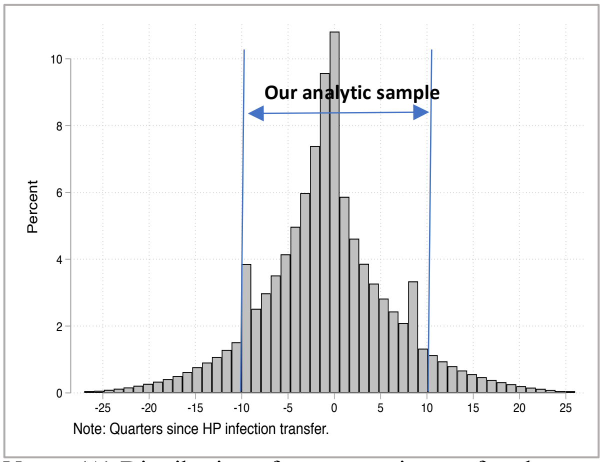

(B) Age distribution of NH residents at the time of hospital transfer.

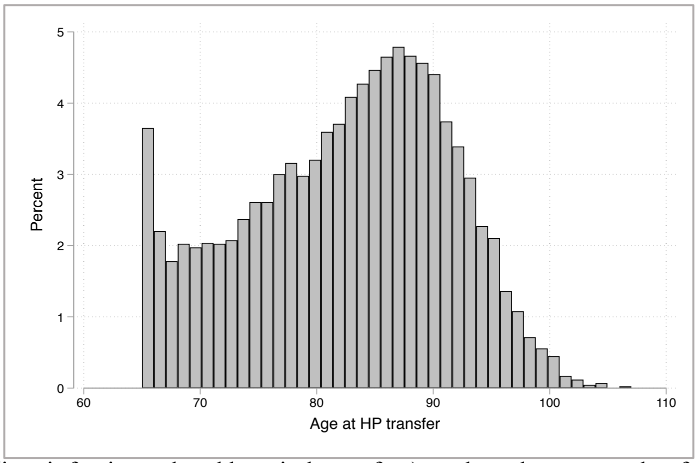

***Notes*: (A)** Distribution of quarters prior or after the event (i.e., infection-related hospital transfers) are based on a sample of nursing home residents who had one infection-related transfer, stayed at the hospital for at most 14 days, were followed at least two quarters prior and four or more after their infection-related transfer. Our final analytical sample on which regressions are estimated is limited to residents followed up to ten quarters prior to or after the infection-related hospitalization transfer to limit possible secular trends prior hospitalization. **(B)** Age distribution of NH residents in our final analytical sample.

© 2021 Gracner et al. *JAMA Network Open*.

eFigure 3. Share of NH residents dying after the infection-related hospital transfer.

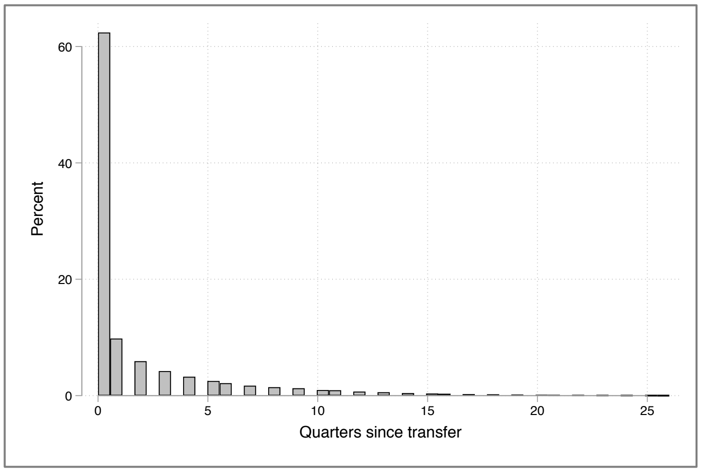

Notes: Share of NH residents dying by quarters since their infection-related hospitalization.

© 2021 Gracner et al. *JAMA Network Open*.

eFigure 4: Change in cognitive function before and after infection-related hospitalization, stratified by age and infection-related condition at transfer (sepsis vs other).

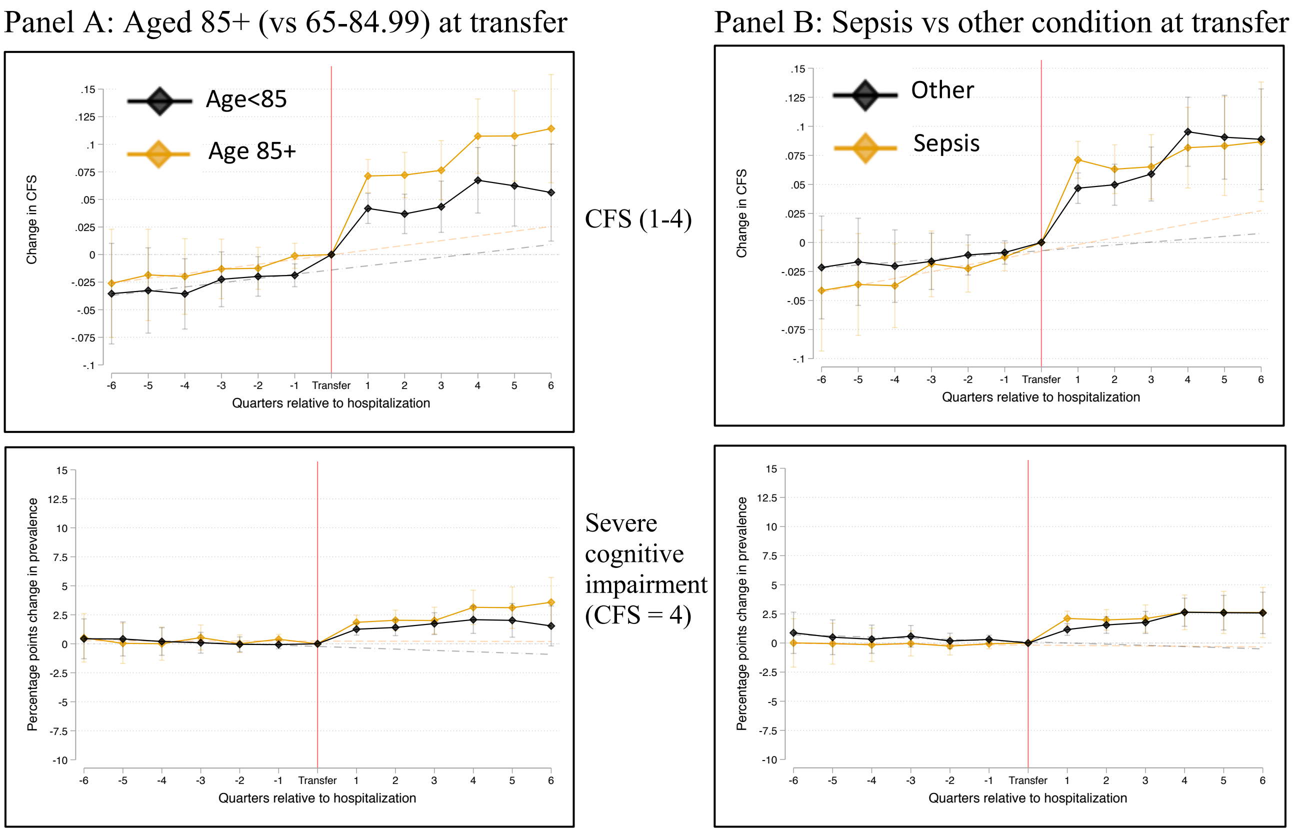

Notes: Changes in cognitive outcomes over time by subgroups. Estimated models are identical to those in Figure 2, except that here each model is estimated for each subgroup. Panel A, older or younger than 85 years; Panel B residents hospitalized due to sepsis vs other infection-related condition. See Figure 2 caption for further details.

© 2021 Gracner et al. *JAMA Network Open*.

eFigure 5: Change in delirium symptoms before and after InfHosp.

(A) Delirium symptoms fluctuate

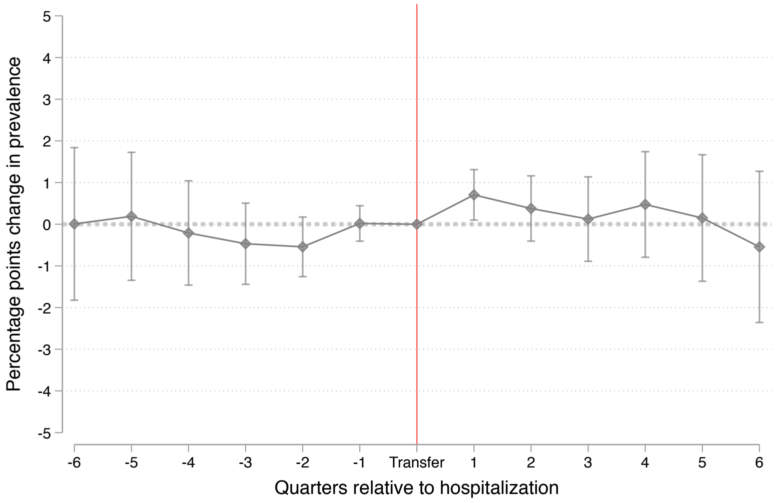

(B) Any delirium symptoms (either fluctuating or continuously present)

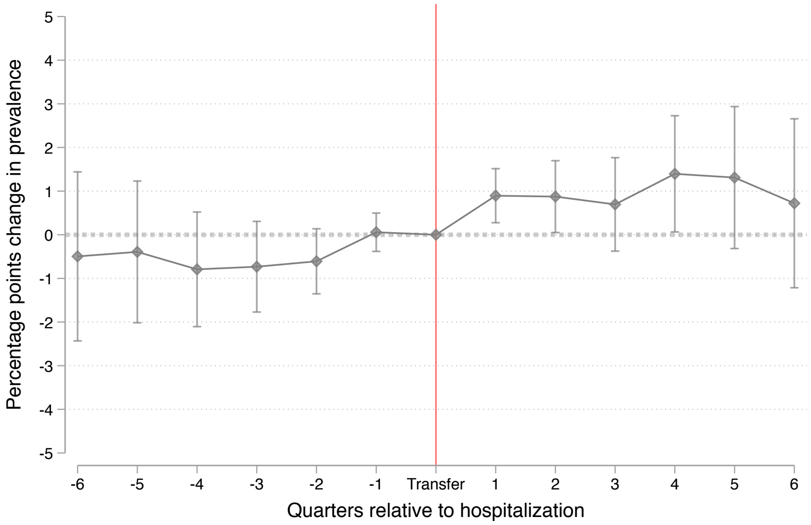

Notes: **(A)** Delirium defined as any acute change or fluctuation in delirium symptoms, inattention, and either disorganized thinking or an altered level of consciousness, or if there was evidence of an acute change in mental status from the resident's baseline. **(B)** Delirium defined as any delirium symptoms that fluctuate *or* are continuously present, such as inattention, and either disorganized thinking or an altered level of consciousness, or if there was evidence of an acute change in mental status from the resident's baseline. 95% confidence intervals are reported.

© 2021 Gracner et al. *JAMA Network Open*.

eTable 1. Characteristics of those surviving at least one year after, and those dying within the first or three quarters of infection-related hospitalization.

Variables evaluated in one quarter pre-InfHosp	Alive 4 + quarters after InfHosp (mean %)	Died in the quarter of InfHosp (mean %)	Died within 3 quarters of InfHosp (mean %)
Age 60-70	11.0%	6.9%	6.6%
Age 71-80	26.6%	22.6%	22.4%
Age 81-90	43.0%	45.5%	45.5%
Age 90+	19.4%	25.0%	25.4%
Female	70.9%	64.1%	63.9%
White	82.4%	82.4%	82.4%
African American	10.6%	10.8%	10.7%
Asian	1.7%	1.9%	2.0%
Hispanic	3.7%	3.5%	3.5%
American Indian/Alaska Native	0.9%	0.8%	0.8%
Pacific Islander	0.7%	0.6%	0.6%
Diabetes Mellitus	54.2%	55.5%	55.0%
Stroke or TIA	39.9%	41.0%	40.7%
Hypertension	78.9%	78.6%	78.3%
Depression	54.0%	50.3%	49.8%
Hip fracture	1.9%	2.0%	2.0%
Shortness of breath	12.1%	14.7%	14.4%
COPD	45.1%	49.5%	48.8%
CHF	59.9%	64.7%	64.1%
AD/ADRD	73.4%	74.8%	75.1%
CFS (1-4)	2.17	2.39	2.42
Severe impairment (1 if CFS = 4, 0 oth.)	9.0%	13.7%	14.4%
% of InfHosp transfers due to sepsis	40.9%	67.0%	60.6%

Notes: Mean values of NH residents' characteristics are reported, as measured in one quarter before infection-related hospital transfer.

eTable 2. Association between infection-related hospitalization and cognitive function over time.

Panel A: Event study approach using quarterly indicators (supporting Figure 2)

Outcome Variable:	**CFS (1-4)**	**CFS = 4**
Approach:	**OLS**	**LMP**
	**(1)**	**(3)**
Quarters relative to transfer (t=0)		
**t-6**	-0.030	0.005
	(-0.064 - 0.004)	(-0.009 - 0.018)
**t-5**	-0.025	0.003
	(-0.053 - 0.004)	(-0.009 - 0.014)
**t-4**	-0.027**	0.001
	(-0.051 - -0.003)	(-0.008 - 0.011)
**t-3**	-0.017	0.003
	(-0.036 - 0.002)	(-0.004 - 0.010)
**t-2**	-0.015**	-0.000
	(-0.029 - -0.002)	(-0.005 - 0.005)
**t-1**	-0.010***	0.002
	(-0.018 - -0.002)	(-0.001 - 0.004)
**t+1**	0.057***	0.016***
	(0.047 - 0.067)	(0.012 - 0.020)
**t+2**	0.055***	0.017***
	(0.042 - 0.069)	(0.012 - 0.023)

© 2021 Gracner et al. *JAMA Network Open*.

**t+3**	0.062***	0.019***
	(0.044 - 0.079)	(0.012 - 0.026)
**t+4**	0.090***	0.026***
	(0.067 - 0.112)	(0.017 - 0.036)
**t+5**	0.087***	0.026***
	(0.060 - 0.115)	(0.014 - 0.038)
**t+6**	0.088***	0.026***
	(0.054 - 0.121)	(0.012 - 0.040)
**All t<-6**	-0.027	0.010
	(-0.068 - 0.015)	(-0.007 - 0.027)
**All t>6**	0.091***	0.028***
	(0.050 - 0.132)	(0.011 - 0.044)
**2012**[a)]	0.066***	0.028***
(vs 2011)	(0.043 - 0.089)	(0.019 - 0.038)
**2013**	0.146***	0.055***
	(0.103 - 0.188)	(0.037 - 0.073)
**2014**	0.216***	0.077***
	(0.155 - 0.278)	(0.051 - 0.103)
**2015**	0.280***	0.099***
	(0.199 - 0.362)	(0.065 - 0.134)
**2016**	0.342***	0.123***
	(0.240 - 0.444)	(0.080 - 0.165)
**2017**	0.425***	0.146***
	(0.303 - 0.547)	(0.095 - 0.197)
**April - June**[b)]	0.023***	0.006***
(vs Jan-March)	(0.016 - 0.029)	(0.003 - 0.009)
**July - Sept**	0.035***	0.011***
	(0.024 - 0.046)	(0.006 - 0.015)
**Oct - Dec**	0.045***	0.017***
	(0.029 - 0.061)	(0.010 - 0.023)
**Age: 70-74**[c)]	-0.116***	-0.024***
	(-0.145 - -0.087)	(-0.036 - -0.012)
**Age: 75-79**	-0.184***	-0.049***
	(-0.223 - -0.145)	(-0.064 - -0.033)
**Age: 80 - 84**	-0.210***	-0.052***
	(-0.257 - -0.163)	(-0.072 - -0.033)
**Age: 85 - 89**	-0.176***	-0.046***
	(-0.228 - -0.124)	(-0.068 - -0.025)
**Age: 90 - 94**	-0.106***	-0.026**
	(-0.163 - -0.049)	(-0.050 - -0.003)
**Age: 94-99**	-0.016	-0.019
	(-0.080 - 0.049)	(-0.046 - 0.007)
**Age: 100+**	0.096**	-0.017
	(0.004 - 0.187)	(-0.054 - 0.020)
**N of chronic conditions: 1**	-0.002	0.001
	(-0.023 - 0.019)	(-0.010 - 0.011)
**N of chronic conditions: 2**	-0.020	-0.001
	(-0.045 - 0.005)	(-0.013 - 0.012)
**N of chronic conditions: 3**	-0.061***	-0.015**
	(-0.089 - -0.033)	(-0.029 - -0.002)
**N of chronic conditions: 4**	-0.139***	-0.031***
	(-0.173 - -0.105)	(-0.047 - -0.016)
**CHF**	-0.003	-0.002
	(-0.022 - 0.017)	(-0.010 - 0.006)
**COPD**	-0.002	-0.004
	(-0.025 - 0.022)	(-0.014 - 0.005)
**Lung cancer**	0.009	-0.024**
	(-0.080 - 0.097)	(-0.045 - -0.003)
**Colorectal cancer**	0.048	-0.027*

© 2021 Gracner et al. *JAMA Network Open*.

	(-0.049 - 0.145)	(-0.058 - 0.004)
**Ischemic heart disease**	-0.019	-0.006
	(-0.044 - 0.006)	(-0.016 - 0.004)
**Years with AD/ADRD 1-3 [d)]**	0.074***	-0.026***
	(0.053 - 0.095)	(-0.032 - -0.019)
**Years with AD/ADRD 4-6**	0.124***	-0.017***
	(0.097 - 0.150)	(-0.026 - -0.007)
**Years with AD/ADRD 7-10**	0.168***	0.002
	(0.135 - 0.200)	(-0.011 - 0.014)
**Years with AD/ADRD 10-14**	0.170***	0.032***
	(0.129 - 0.211)	(0.014 - 0.050)
**Years with AD/ADRD 15+**	0.128***	0.036**
	(0.068 - 0.188)	(0.008 - 0.065)
**Hip fracture**	0.023	0.003
	(-0.006 - 0.051)	(-0.007 - 0.012)
**Shortness of breath lying or sitting**	0.027***	0.004*
	(0.016 - 0.038)	(-0.000 - 0.008)
**Observations**	266,196	266,196

Panel B: Event study approach imposing linear trend in outcomes pre-hospitalization.

Outcome Variable:	**CFS (1-4)**	**CFS = 4**
Approach:	**OLS**	**LMP**
	**(1)**	**(3)**
Pre-InfHosp linear trend	0.022***	0.006***
	(0.020 - 0.024)	(0.005 - 0.006)
Quarter effects relative to linear pre-InfHosp trend		
**t+1**	0.056***	0.016***
	(0.047 - 0.066)	(0.012 - 0.020)
**t+2**	0.050***	0.018***
	(0.039 - 0.062)	(0.014 - 0.023)
**t+3**	0.052***	0.020***
	(0.039 - 0.065)	(0.015 - 0.025)
**t+4**	0.076***	0.028***
	(0.061 - 0.091)	(0.022 - 0.034)
**t+5**	0.069***	0.029***
	(0.052 - 0.086)	(0.022 - 0.035)
**t+6**	0.065***	0.029***
	(0.046 - 0.084)	(0.021 - 0.037)
**All t<-6**	0.013**	0.005**
	(0.000 - 0.025)	(0.001 - 0.010)
**All t>6**	0.060***	0.032***
	(0.038 - 0.082)	(0.023 - 0.041)
**2012[a)]**	-0.004	0.004*
(vs 2011)	(-0.015 - 0.007)	(-0.001 - 0.009)
**2013**	0.004	0.006**
	(-0.008 - 0.016)	(0.001 - 0.012)
**2014**	0.004	0.004
	(-0.008 - 0.016)	(-0.002 - 0.009)
**2015**	-0.003	0.002
	(-0.015 - 0.009)	(-0.003 - 0.007)
**2016**	-0.012**	0.001
	(-0.023 - -0.001)	(-0.004 - 0.006)
**April - June[b)]**	0.005**	-0.000
(vs Jan-March)	(0.001 - 0.009)	(-0.002 - 0.001)
**July - Sept**	-0.001	-0.001
	(-0.005 - 0.004)	(-0.003 - 0.001)
**Oct - Dec**	-0.008***	-0.001
	(-0.012 - -0.004)	(-0.003 - 0.000)
**Age: 70-74[c)]**	-0.116***	-0.024***

© 2021 Gracner et al. *JAMA Network Open.*

	(-0.145 - -0.087)	(-0.036 - -0.012)
**Age: 75-79**	-0.184***	-0.049***
	(-0.223 - -0.145)	(-0.064 - -0.033)
**Age: 80 - 84**	-0.210***	-0.052***
	(-0.257 - -0.163)	(-0.072 - -0.033)
**Age: 85 - 89**	-0.176***	-0.046***
	(-0.228 - -0.124)	(-0.068 - -0.025)
**Age: 90 - 94**	-0.106***	-0.026**
	(-0.163 - -0.049)	(-0.050 - -0.003)
**Age: 94-99**	-0.016	-0.019
	(-0.080 - 0.049)	(-0.046 - 0.007)
**Age: 100+**	0.096**	-0.017
	(0.005 - 0.187)	(-0.054 - 0.020)
**N of chronic conditions: 1**	-0.002	0.001
	(-0.023 - 0.019)	(-0.010 - 0.011)
**N of chronic conditions: 2**	-0.020	-0.001
	(-0.045 - 0.005)	(-0.013 - 0.012)
**N of chronic conditions: 3**	-0.061***	-0.016**
	(-0.089 - -0.033)	(-0.029 - -0.002)
**N of chronic conditions: 4**	-0.139***	-0.031***
	(-0.173 - -0.105)	(-0.047 - -0.016)
**CHF**	-0.002	-0.002
	(-0.022 - 0.017)	(-0.010 - 0.006)
**COPD**	-0.002	-0.004
	(-0.025 - 0.022)	(-0.014 - 0.005)
**Lung cancer**	0.009	-0.024**
	(-0.080 - 0.097)	(-0.045 - -0.003)
**Colorectal cancer**	0.048	-0.027
	(-0.049 - 0.145)	(-0.058 - 0.004)
**Ischemic heart disease**	-0.019	-0.006
	(-0.044 - 0.006)	(-0.016 - 0.004)
**Years with AD/ADRD 1-3 [d)]**	0.074***	-0.026***
	(0.053 - 0.095)	(-0.032 - -0.019)
**Years with AD/ADRD 4-6**	0.124***	-0.017***
	(0.097 - 0.150)	(-0.026 - -0.007)
**Years with AD/ADRD 7-10**	0.168***	0.002
	(0.135 - 0.200)	(-0.011 - 0.014)
**Years with AD/ADRD 10-14**	0.170***	0.032***
	(0.129 - 0.211)	(0.014 - 0.050)
**Years with AD/ADRD 15+**	0.128***	0.036**
	(0.068 - 0.188)	(0.008 - 0.065)
**Hip fracture**	0.023	0.002
	(-0.006 - 0.051)	(-0.007 - 0.012)
**Shortness of breath lying or sitting**	0.027***	0.004
	(0.016 - 0.038)	(-0.000 - 0.008)
**Observations**	266,196	266,196

Notes: a) Reference year is 2011. b) Reference quarter is Jan-March. c) Reference age group is 65-69. d) Reference group are those without ADRD or with 0 years with the disease. Chronic conditions include diabetes, hypertension, obesity and stroke or TIA. All regressions include NH resident indicators. **, and *** refer to a p-value smaller than 0.05 and 0.001, respectively. Robust standard errors are clustered at the nursing home level. 95% confidence intervals are reported in brackets. OLS refers to ordinary least square and LPM to linear probability model estimation approach.

© 2021 Gracner et al. *JAMA Network Open*.

eTable 3. Average and heterogenous effect of infection-related hospitalization on cognitive function by sex, age and AD/ADRD at the time of transfer.

Outcome:	**CFS**	**Severe impairment**
	**(1-4)**	**CFS = 4**
Approach:	**OLS**	**LMP**
	**(1)**	**(3)**
**PANEL A: Average effect**		
**Post Transfer**	0.054***	0.015***
95% CI	(0.044 - 0.063)	(0.011 - 0.019)
**% Change**	2.49%	16.67%
Mean at transfer	2.17	9.00%
**Observations**	266,157	266,157
**PANEL B: Subgroup analyses**		
**i) Female vs male**		
**Post Transfer**	0.048***	0.013***
95% CI	(0.031 - 0.065)	(0.007 - 0.019)
**Post Transfer x Female**	0.008	0.003
95% CI	(-0.012 - 0.028)	(-0.004 - 0.011)
Mean at transfer if male	2.13	7.55%
**ii) Sepsis vs other**		
**Post Transfer**	0.045***	0.010***
95% CI	(0.033 - 0.056)	(0.005 - 0.015)
**Post Transfer x Sepsis**	0.023**	0.012***
95% CI	(0.004 - 0.041)	(0.005 - 0.020)
Mean at transfer for non-sepsis transfer	2.14	7.78%
**iii) 85+ vs younger at transfer**		
**Post Transfer**	0.043***	0.016***
95% CI	(0.031 - 0.055)	(0.011 - 0.021)
**Post Transfer x Age 85+**	0.022**	-0.003
95% CI	(0.004 - 0.040)	(-0.010 - 0.005)
Mean at transfer for age<85	2.06	9.07%
**iv) AD/ADRD vs no AD/ADRD at transfer**		
**Post Transfer**	0.032***	0.004
95% CI	(0.014 - 0.049)	(-0.001 - 0.008)
**Post Transfer x AD/ADRD**	0.029***	0.015***
95% CI	(0.009 - 0.050)	(0.009 - 0.021)
Mean at transfer if no AD/ADRD	1.59	2.48%

Notes: Each regression is adjusted for active diagnoses of hip fracture, shortness of breath, AD/ADRD and years with the disease, number of chronic conditions (1,2,3, or 4), resident's age indicators, calendar quarter indicators, year indicators, and trend before and after 6 quarters after the hospital transfer. All dependent variables except a continuous measure of CFS (1-4) are indicator variables. OLS refers to Ordinary Least Squares and LMP to linear probability model. **, and *** imply p value being smaller than 0.05, and 0.01, respectively. Robust standard errors clustered at the nursing home level are reported. 95% confidence intervals are reported in brackets.

© 2021 Gracner et al. *JAMA Network Open*.

eTable 4. Association between infection-related hospitalization and cognitive function over time, using alternate samples.

Outcome Variable:	CFS (1-4)	Severe impairment (if CFS = 4)
Approach:	OLS	LMP
	(1)	(3)
**Panel A: Sample restricted to 2 or more quarters prior to and after hospitalization**		
Pre-hospitalization linear trend	0.024***	0.006***
	(0.022 - 0.026)	(0.005 - 0.007)
Quarter effects relative to linear pre-InfHosp trend		
**t+1**	0.068***	0.021***
	(0.060 - 0.076)	(0.018 - 0.024)
**t+2**	0.077***	0.026***
	(0.068 - 0.087)	(0.023 - 0.030)
**t+3**	0.069***	0.027***
	(0.058 - 0.080)	(0.023 - 0.031)
**t+4**	0.073***	0.029***
	(0.059 - 0.086)	(0.023 - 0.034)
**t+5**	0.064***	0.028***
	(0.049 - 0.079)	(0.022 - 0.034)
**t+6**	0.058***	0.028***
	(0.041 - 0.075)	(0.021 - 0.035)
**Observations**	349,572	349,572
**Panel B: Sample restricted to at least 6 quarters pre/post InfHosp (i.e. balanced sample)**		
Pre-hospitalization linear trend	0.017***	0.004***
	(0.015 - 0.020)	(0.003 - 0.005)
Quarter effects relative to linear pre-InfHosp trend		
**t+1**	0.059***	0.013***
	(0.047 - 0.070)	(0.009 - 0.018)
**t+2**	0.055***	0.016***
	(0.043 - 0.068)	(0.011 - 0.021)
**t+3**	0.055***	0.017***
	(0.040 - 0.070)	(0.011 - 0.022)
**t+4**	0.068***	0.020***
	(0.051 - 0.085)	(0.014 - 0.027)
**t+5**	0.080***	0.026***
	(0.061 - 0.099)	(0.019 - 0.034)
**t+6**	0.093***	0.030***
	(0.071 - 0.114)	(0.021 - 0.038)
**Observations**	198,883	198,883
**Panel C: Sample includes residents with up to 2 hospitalizations**		
Pre-hospitalization linear trend	0.020***	0.005***
	(0.018 - 0.022)	(0.004 - 0.006)
Quarter effects relative to linear pre-InfHosp trend		
**t+1**	0.055***	0.016***
	(0.047 - 0.062)	(0.013 - 0.019)
**t+2**	0.052***	0.019***
	(0.043 - 0.061)	(0.015 - 0.022)
**t+3**	0.057***	0.020***
	(0.047 - 0.068)	(0.016 - 0.024)
**t+4**	0.078***	0.027***
	(0.066 - 0.090)	(0.022 - 0.032)
**t+5**	0.074***	0.028***
	(0.060 - 0.088)	(0.023 - 0.033)
**t+6**	0.072***	0.029***
	(0.057 - 0.088)	(0.023 - 0.035)
**Observations**	392,312	392,312

© 2021 Gracner et al. *JAMA Network Open*.

Panel D: Sample includes residents who died within the first three quarters of transfer		
Pre-hospitalization linear trend	0.038***	0.009***
	(0.037 - 0.040)	(0.008 - 0.010)
Quarter effects relative to linear pre-InfHosp trend		
**t+1**	0.114***	0.040***
	(0.103 - 0.124)	(0.035 - 0.044)
**t+2**	0.094***	0.034***
	(0.079 - 0.108)	(0.027 - 0.040)
**t+3**	0.104***	0.046***
	(0.083 - 0.126)	(0.036 - 0.055)
**Observations**	390,101	390,101

Notes: Refer to captions from previous tables in terms of regression model approach, as it is the same as for each sample as described for the parametric event study approach. **, and *** refer to a p-value smaller than 0.05 and 0.01, respectively. Robust standard errors are clustered at the nursing home level. 95% confidence intervals are reported in brackets. Other alternative sample selections were chosen not reported here, but conclusions remained unchanged.

eTable 5. Association between infection-related hospitalization and cognitive function over time, using placebo hospitalization dates (4 quarters earlier).

Outcome:	**CFS**	**Severe impairment**
	**(1-4)**	**(if CFS = 4)**
**Post Transfer**	-0.005	-0.000
95% CI	(-0.017 - 0.007)	(-0.004 - 0.003)
Observations	107,722	107,722

Notes: Refer to caption under eTables 2-4 for details. Placebo transfer date is constructed 4 quarters prior to each resident's actual infection-related hospitalization date, and the analytic sample is limited to pre-hospitalization quarters.

eTable 6: Mean CFS scores and severe impairment prevalence at transfer by subgroups.

	**CFS (1-4)**	**Severe impairment (CFS = 4)**
**Female vs Male**	2.18 vs 2.13	9.5% vs 7.55%
**With AD/ADRD vs without**	2.37 vs 1.59	11.2% vs 2.48%
**With sepsis vs other diagnoses**	2.21 vs 2.14	10.58% vs 7.78%
**85+ years old vs younger**	2.29 vs 2.06	8.78% vs 9.07%

© 2021 Gracner et al. *JAMA Network Open*.

eTable 7: ICD-9 infection codes

ICD-9 code	Description
098.0	Gonococcal infection (acute) of lower genitourinary tract
098.10	Gonococcal infection (acute) of upper genitourinary tract site unspecified
098.19	Other Gonococcal infection (acute) of upper genitourinary tract
596.81	Infection of Cystostomy
599.0	Urinary tract infection site not specified
996.64	Infection and inflammation due to indwelling urinary catheter
997.5	Urinary complications, not elsewhere classified (ICD-9 book doesn't list infection as an option, but it may be used for Postprocedural UTI)
003.22	Salmonella pneumonia
039.1	Pulmonary actinomycotic infection
041.00	Streptococcus infection in conditions classified elsewhere and of unspecified site, streptococcus, unspecified
041.01	Streptococcus infection in conditions classified elsewhere and of unspecified site, streptococcus, group A
041.02	Streptococcus infection in conditions classified elsewhere and of unspecified site, streptococcus, group B
041.03	Streptococcus infection in conditions classified elsewhere and of unspecified site, streptococcus, group C
041.04	Streptococcus infection in conditions classified elsewhere and of unspecified site, streptococcus, group D [Enterococcus]
041.05	Streptococcus infection in conditions classified elsewhere and of unspecified site, streptococcus, group G
041.09	Streptococcus infection in conditions classified elsewhere and of unspecified site, other streptococcus
041.10	Staphylococcus infection in conditions classified elsewhere and of unspecified site, staphylococcus, unspecified
041.11	Methicillin susceptible Staphylococcus aureus in conditions classified elsewhere and of unspecified site
041.12	Methicillin resistant Staphylococcus aureus in conditions classified elsewhere and of unspecified site
041.19	Staphylococcus infection in conditions classified elsewhere and of unspecified site, other staphylococcus
041.2	Pneumococcus infection NOS
041.3	Klebsiella pneumonia
041.4	Escherichia coli [e. coli] infection in conditions classified elsewhere and of unspecified site
041.41	Shiga toxin-producing Escherichia coli [E. coli] (STEC) O157
041.42	Other specified Shiga toxin-producing Escherichia coli [E. coli] (STEC)
041.43	Shiga toxin-producing Escherichia coli [E. coli] (STEC), unspecified
041.49	Other and unspecified Escherichia coli [E. coli]
041.5	Hemophilus influenza (H. Influenzae) infection in conditions classified elsewhere and of unspecific site
041.6	Proteus (mirabilis) (morganii) infection in conditions classified elsewhere and of unspecified site
041.7	Pseudomonas infection in conditions classified elsewhere and of unspecified site
041.81	Other specified bacterial infections in conditions classified elsewhere and of unspecified site, mycoplasma
041.82	Bacteroides fragilis
041.83	Other specified bacterial infections in conditions classified elsewhere and of unspecified site, Clostridium perfringens
041.84	Other specified bacterial infections in conditions classified elsewhere and of unspecified site, other anaerobes
041.85	Other specified bacterial infections in conditions classified elsewhere and of unspecified site, other gram-negative organisms
041.89	Other specified bacterial infections in conditions classified elsewhere and of unspecified site, other specified bacteria
041.9	Bacterial infection, unspecified, in conditions classified elsewhere and of unspecified site
482.83	Pneumonia due to other Gram-negative bacteria
055.1	Postmeasles pneumonia
073.0	Ornithosis with pneumonia
098.6	Gonococcal infection of pharynx
475	Peritonsillar abscess
478.21	Cellulitis diffuse with lymphangitis of nasopharynx or pharynx
481	Pneumococcal pneumonia (streptococcus pneumoniae pneumonia)
482.0	Pneumonia due to KLEBSIELLA PNEUMONIAE

© 2021 Gracner et al. *JAMA Network Open*.

482.1	Pneumonia due to PSEUDOMONAS
482.2	Pneumonia due to HEMOPHILUS INFLUENZAE (H. INFLUENZAE)
482.30	Pneumonia due to Streptococcus unspecified
482.31	Pneumonia due to Streptococcus Group A
482.32	Pneumonia due to Streptococcus Group B
482.39	Pneumonia due to other Streptococcus
482.40	Pneumonia due to Staphylococcus unspecified
482.41	Methicillin susceptible pneumonia due to staphylococcus aureus
482.42	Methicillin resistant pneumonia due to staphylococcus aureus
482.49	Other staphylococcus pneumonia
482.81	Pneumonia due to anaerobes
482.82	Pneumonia due to Escherichia Coli (E. Coli)
482.83	Pneumonia due to other Gram-negative bacteria
482.84	Pneumonia due to Legionnaires' disease
482.89	Pneumonia due to other specified bacteria
482.9	Bacterial pneumonia unspecified
483.0	Pneumonia due to mycoplasma pneumonia
483.1	Pneumonia due to chlamydia
483.8	Pneumonia due to other specified organism
484.5	Pneumonia in Anthrax
513.0	Abscess of lung
513.1	Abscess of mediastinum
997.31	Ventilator associated pneumonia
997.32	Postprocedural aspiration pneumonia
999.31	Other and unspecified infection due to central venous catheter
999.32	Bloodstream infection due to central venous catheter
999.33	Local infection due to central venous catheter
996.62	Infection and inflammation due to other vascular devise implant and graft, including arterial, dialysis, and peripheral vascular catheter
003.0	Salmonella gastroenteritis
098.49	Other Gonococcal infection of eye
098.52	Gonococcal bursitis
098.59	Other Gonococcal infection of joint
098.7	Gonococcal infection of anus and rectum
098.89	Gonococcal infection of other specified sites or Gonococcal Septicemia
376.01	Orbital cellulitis
478.21	Cellulitis of pharynx or nasopharynx
478.71	Cellulitis and perichondritis of larynx
528.3	Cellulitis and abscess of oral soft tissues
614.3	Acute parametritis and pelvic cellulitis
680.0	Carbuncle and furuncle of face
680.1	Carbuncle and furuncle of neck
680.2	Carbuncle and furuncle of trunk
680.3	Carbuncle and furuncle of upper arm and forearm
680.4	Carbuncle and furuncle of hand
680.5	Carbuncle and furuncle of buttock
680.6	Carbuncle and furuncle of leg
680.7	Carbuncle and furuncle of foot
680.8	Carbuncle and furuncle of head and scalp
680.9	Carbuncle and furuncle unspecified site
681.00	Cellulitis and abscess of finger, unspecified
681.10	Cellulitis and abscess of toe, unspecified
681.9	Cellulitis and abscess of unspecified digit
682.0	Cellulitis and abscess of face
682.1	Cellulitis and abscess of neck
682.2	Cellulitis and abscess of trunk
682.3	Cellulitis and abscess of upper arm and forearm
682.4	Cellulitis and abscess of hand, except fingers and thumb
682.5	Cellulitis and abscess of buttock
682.6	Cellulitis and abscess of leg, except foot
682.7	Cellulitis and abscess of foot, except toes
682.8	Cellulitis and abscess of other specified sites
682.9	Cellulitis and abscess of unspecified sites
684	Impetigo
686.00	Pyoderma, unspecified
686.01	Pyoderma gangrenosum

© 2021 Gracner et al. *JAMA Network Open*.

686.1	Pyogenic granuloma of skin and subcutaneous tissue
686.09	Other Pyoderma
686.8	Local skin infection
686.9	Unspecified local infection of skin and subcutaneous tissue
728.0	Infective myositis
728.86	Necrotizing fasciitis
730.0	Osteomyelitis acute
730.01	Osteomyelitis shoulder
730.02	Osteomyelitis upper arm
730.03	Osteomyelitis forearm
730.04	Osteomyelitis hand
730.05	Osteomyelitis pelvis & thigh
730.06	Osteomyelitis lower leg
730.07	Osteomyelitis ankle & foot
730.08	Osteomyelitis other
730.09	Osteomyelitis multiple sites
730.1	Osteomyelitis chronic
730.2	Osteomyelitis unspecified
730.3	periostitis
730.8	Other Bone infection
730.9	Unspecified infection of bone
996.60	Infection and inflammatory reaction due to unspecified device, implant, and graft
996.61	Infection and inflammatory reaction due to cardiac device, implant, and graft
996.63	Infection and inflammatory reaction due to nervous system device, implant, and graft
996.65	Infection and inflammatory reaction due to other genitourinary device, implant, and graft
996.66	Infection and inflammatory reaction due to internal joint prosthesis
996.67	Infection and inflammatory reaction due to other internal orthopedic device, implant, and graft
996.68	Infection and inflammatory reaction due to peritoneal dialysis catheter
996.69	Infection and inflammatory reaction due to other internal prosthetic device, implant, and graft
998.51	Infected postoperative seroma
998.59	Other postoperative infection
001.0	Cholera due to Vibrio cholerae 01, biovar cholerae
001.1	Cholera due to Vibrio cholerae 01, biovar eltor
001.9	Cholera, unspecified
002.0	Typhoid fever, unspecified
002.1	Paratyphoid fever A
002.3	Paratyphoid fever C
002.9	Paratyphoid fever, unspecified
003.0	Salmonella enteritis
003.1	Salmonella sepsis
003.20	Localized salmonella infection, unspecified
003.21	Salmonella meningitis
003.22	Salmonella pneumonia
003.23	Salmonella arthritis
003.24	Salmonella osteomyelitis
003.29	Salmonella with other localized infection
003.8	Other specified salmonella infections
003.9	Salmonella infection, unspecified
008.00	Other intestinal Escherichia coli infections
008.01	Enteropathogenic Escherichia coli infection
008.02	Enterotoxigenic Escherichia coli infection
008.03	Enteroinvasive Escherichia coli infection
008.04	Enterohemorrhagic Escherichia coli infection
008.09	Other intestinal Escherichia coli infections
008.1	Other specified bacterial intestinal infections
008.2	Other specified bacterial intestinal infections
008.3	Other specified bacterial intestinal infections
008.41	Other specified bacterial intestinal infections
008.42	Other specified bacterial intestinal infections
008.43	Campylobacter enteritis
008.44	Enteritis due to Yersinia enterocolitica
008.45	Enterocolitis due to Clostridium difficile, recurrent
008.46	Other specified bacterial intestinal infections
008.47	Other specified bacterial intestinal infections

© 2021 Gracner et al. *JAMA Network Open*.

008.49	Other specified bacterial intestinal infections
008.5	Bacterial intestinal infection, unspecified
036.2	Meningococcal septicemia
038.0	Streptococcal septicemia
038.10	Staphylococcal septicemia NOS
038.11	MSSA septicemia
038.12	MRSA septicemia
038.19	Other staphylococcal septicemia
038.2	Pneumococcal septicemia
038.3	Anaerobic septicemia
038.40	Gram-negative septicemia NOS
038.41	H. influenzae septicemia
038.42	E. coli septicemia
038.43	Pseudomonas septicemia
038.44	Serratia septicemia
038.49	Gram-neg septicemia NEC
038.8	Septicemia NEC
038.9	Septicemia NOS
785.52	Septic shock
790.7	Bacteremia
995.90	SIRS (Systemic inflammatory response syndrome)
995.91	Sepsis
995.92	Severe sepsis
020.0	Plague
020.1	Plague
020.2	Septicemic plague
020.3	Plague
020.4	Plague
020.5	Plague
020.8	Plague
020.9	Plague
021	Tularemia
022	Anthrax
023	Brucellosis
024	Glanders
025	Meliodiosis
026	Rat-bite fever
027	Other bacterial zoonoses
032	Pharyngeal diphtheria
033	Whooping cough
034	Streptococcal throat/scarlet fever
037	Tetanus
040	Other bacterial diseases
091	Early syphilis, symptomatic
092	Early syphilis, latent
095	Other forms of late syphilis, with symptoms
096	Late syphilis, latent
097	Late syphilis, unspecified
102	Yaws
103	Pinta
104	Nonvenereal syphilis
590	Nonobstructive reflux-associated chronic pyelonephritis
597	Urethral abscess
601	Acute prostatitis
615	Acute inflammatory disease of uterus
616	Inflammatory disease of cervix uteri
711.0	Staphylococcal arthritis, unspecified joint
V09.51	infection with microorganisms resistant to multiple quinolones and fluoroquinolones
V09.80	infection with microorganisms resistant to other specified drugs without resistance to multiple drugs
V09.81	infection with microorganisms resistant to other specified drugs with resistance to multiple drugs
V09.90	infection with drug-resistant microorganisms unspecified without multiple drug resistance
V09.91	infection with drug-resistant microorganisms unspecified with multiple drug resistance
038.12	methicillin resistant staphylococcus aureus septicemia

© 2021 Gracner et al. *JAMA Network Open*.

041.12	methicillin resistant staphylococcus infection in conditions classified elsewhere and of unspecified site staphylococcus aureus
482.42	methicillin resistant pneumonia due to staphylococcus aureus
V09	infection with drug-resistant microorganisms
V09.0	infection with microorganisms resistant to penicillins
V09.1	infection with microorganisms resistant to cephalosporins and other b-lactam antibiotics
V09.2	infection with microorganisms resistant to macrolides
V09.3	infection with microorganisms resistant to tetracyclines
V09.4	infection with microorganisms resistant to aminoglycosides
V09.5	infection with microorganisms resistant to quinolones and fluoroquinolones
V09.6	infection with microorganisms resistant to sulfonamides
V09.8	infection with microorganisms resistant to other specified drugs
V09.80	infection with microorganisms resistant to other specified drugs without resistance to multiple drugs
V09.81	infection with microorganisms resistant to other specified drugs with resistance to multiple drugs
V09.9	infection with drug-resistant microorganisms unspecified
V09.90	infection with drug-resistant microorganisms unspecified without multiple drug resistance
V09.91	infection with drug-resistant microorganisms unspecified with multiple drug resistance
460	Acute nasopharyngitis
461.0	Acute maxillary sinusitis
461.1	Acute frontal sinusitis
461.2	Acute ethmoidal sinusitis
461.3	Acute sphenoidal sinusitis
461.8	Other acute sinusitis
461.9	Acute sinusitis unspecified
462	Acute pharyngitis
463	Acute tonsillitis
464	Acute laryngitis and tracheitis
464.00	Acute laryngitis without obstruction
464.01	Acute laryngitis with obstruction
464.10	Acute tracheitis without obstruction
464.11	Acute tracheitis with obstruction
464.20	Acute laryngotracheitis without obstruction
464.21	Acute laryngotracheitis with obstruction
464.30	Acute epiglottitis without obstruction
464.31	Acute epiglottitis with obstruction
464.50	Supraglottitis unspecified without obstruction
464.51	Supraglottitis unspecified with obstruction
465.0	Acute laryngopharyngitis
465.8	Acute upper respiratory infections of other multiple sites
465.9	Acute upper respiratory infections of unspecific site
466.0	Acute bronchitis
484.8	Pneumonia in infectious diseases classified elsewhere
486	Pneumonia organism unspecified
491.22	Acute bronchitis with COPD
999.34	Acute infection following transition, infusion, or injections of blood and blood products
999.39	Infection following other infusion, injection, transfusion, or vaccination (non-specific code)
008.8	Other specified intestinal infections
009.0	Infectious gastroenteritis and colitis, unspecified
009.1	Infectious gastroenteritis and colitis, unspecified
009.2	Infectious gastroenteritis and colitis, unspecified
009.3	Infectious gastroenteritis and colitis, unspecified
683	Acute lymphadenitis, unspecified

© 2021 Gracner et al. *JAMA Network Open*.

eTable 8: ICD-10 infection codes

ICD-10 code	Description
A00.0	Cholera due to Vibrio cholerae 01, biovar cholerae
A00.1	Cholera due to Vibrio cholerae 01, biovar eltor
A00.9	Cholera, unspecified
A01.00	Typhoid fever, unspecified
A01.05	typhoid osteomyelitis
A01.1	Paratyphoid fever A
A01.3	Paratyphoid fever C
A01.4	Paratyphoid fever, unspecified
A02.0	Salmonella enteritis
A02.1	Salmonella sepsis
A02.20	Localized salmonella infection, unspecified
A02.21	Salmonella meningitis
A02.22	Salmonella pneumonia
A02.23	Salmonella arthritis
A02.24	Salmonella osteomyelitis
A02.25	salmonella pyelonephritis
A02.29	Salmonella with other localized infection
A02.8	Other specified salmonella infections
A02.9	Salmonella infection, unspecified
A04.0	Enteropathogenic Escherichia coli infection
A04.1	Enterotoxigenic Escherichia coli infection
A04.2	Enteroinvasive Escherichia coli infection
A04.3	Enterohemorrhagic Escherichia coli infection
A04.4	Other intestinal Escherichia coli infections
A04.5	Campylobacter enteritis
A04.6	Enteritis due to Yersinia enterocolitica
A04.71	Enterocolitis due to Clostridium difficile, recurrent
A04.8	Other specified bacterial intestinal infections
A04.9	Bacterial intestinal infection, unspecified
A20.0	Plague
A20.1	Plague
A20.2	Plague
A20.7	Septicemic plague
A20.8	Plague
A20.9	Plague
A21.0	Tularemia
A21.1	Tularemia
A21.2	Tularemia
A21.3	Tularemia
A21.7	Tularemia
A21.8	Tularemia
A21.9	Tularemia
A22.0	Anthrax
A22.1	Anthrax
A22.2	Anthrax
A22.7	Anthrax
A22.8	Anthrax
A22.9	Anthrax
A23.0	Brucellosis
A23.1	Brucellosis
A23.2	Brucellosis
A23.3	Brucellosis
A23.8	Brucellosis
A23.9	Brucellosis
A24.0	Glanders
A24.3	Melioidosis
A24.9	Melioidosis
A25.0	Spirillosis
A27.0	Leptospirosis
A32.7	Listerial sepsis
A35	Tetanus
A36.0	Diphteria
A37.00	whooping cough due to bordetella pertussis without pneumonia

© 2021 Gracner et al. *JAMA Network Open*.

A37.01	whooping cough due to bordetella pertussis with pneumonia
A37.10	whooping cough due to bordetella parapertussis without pneumonia
A37.11	whooping cough due to bordetella parapertussis with pneumonia
A37.80	whooping cough due to other bordetella species without pneumonia
A37.81	whooping cough due to other bordetella species with pneumonia
A37.90	whooping cough, unspecified species without pneumonia
A37.91	whooping cough, unspecified species with pneumonia
A39.4	Meningococcal sepsis
A40.0	sepsis due to streptococcus, group a
A40.1	sepsis due to streptococcus, group b
A40.8	other streptococcal sepsis
A41.81	sepsis due to enterococcus
A40.3	sepsis due to streptococcus pneumoniae
A40.9	Streptococcal sepsis, unspecified
A41.01	MSSA sepsis
A41.02	MRSA sepsis
A41.1	Other staphylococcal sepsis
A41.2	Staphylococcal sepsis (unspecified)
A41.3	Hemophilus influenzae
A41.4	Sepsis due to anaerobes
A41.50	Gram-negative sepsis, unspecified
A41.51	Sepsis due to e. coli
A41.52	Sepsis due to pseudomonas
A41.53	Sepsis due to serratia
A41.59	Other Gram-negative sepsis
A41.89	Other specified sepsis
A41.9	Unspecified sepsis
A42.0	Pulmonary actinomycotic infection
A48.0	Other bacterial deseases
A48.1	Pneumonia due to Legionnaires' disease
A49.01	methicillin susceptible staphylococcus aureus infection, unspecified site
A49.02	methicillin resistant staphylococcus aureus infection, unspecified site
A49.1	streptococcal infection, unspecified site
A49.3	Mycoplasma infection, unspecified site
A49.8	other bacterial infections of unspecified site
A49.9	bacterial infection, unspecified
A51.0	Primary genital syphilis
A51.5	Early syphilis, latent
A52.71	Late syphilitic oculopathy
A52.8	Late syphilis, latent
A52.9	Late syphilis, unspecified
A54.00	Gonococcal infection (acute) of lower genitourinary tract
A54.29	Other gonococcal genitourinary infections
A54.39	Other Gonococcal infection of eye
A54.43	gonococcal osteomyelitis
A54.49	Gonococcal infection of other musculoskeletal tissue
A54.5	Gonococcal infection of pharynx
A54.6	Gonococcal infection of anus and rectum
A54.9	Gonococcal infection of other specified sites
A65	Other spirochetal infection
A66.0	Initial lesions of yaws
A67.0	Primary lesions of pinta
A70	Chlamydia psittaci infections
B05.2	Postmeasles pneumonia
B95.0	Streptococcus, group A, as the cause of diseases classified elsewhere
B95.1	Streptococcus, group B, as the cause of diseases classified elsewhere
B95.2	Enterococcus as the cause of diseases classified elsewhere
B95.3	Streptococcus pneumoniae as the cause of diseases classified elsewhere
B95.4	Other streptococcus as the cause of diseases classified elsewhere
B95.5	Unspecified streptococcus as the cause of diseases classified elsewhere
B95.61	Methicillin susceptible Staphylococcus aureus infection as the cause of diseases classified elsewhere
B95.62	Methicillin resistant Staphylococcus aureus infection as the cause of diseases classified elsewhere
B95.7	Other staphylococcus as the cause of diseases classified elsewhere
B95.8	Unspecified staphylococcus as the cause of diseases classified elsewhere
B96.0	Mycoplasma pneumoniae [M. pneumoniae] as the cause of diseases classified elsewhere

© 2021 Gracner et al. *JAMA Network Open*.

B96.1	Klebsiella pneumoniae [K. pneumoniae] as the cause of diseases classified elsewhere
B96.20	unspecified escherichia coli [e. coli] as the cause of diseases classified elsewhere
B96.21	Shiga toxin-producing Escherichia coli [E. coli] (STEC) O157 as the cause of diseases classified elsewhere
B96.22	Other specified Shiga toxin-producing Escherichia coli [E. coli] (STEC) as the cause of diseases classified elsewhere
B96.23	Unspecified Shiga toxin-producing Escherichia coli [E. coli] (STEC) as the cause of diseases classified elsewhere
B96.29	Other Escherichia coli [E. coli] as the cause of diseases classified elsewhere
B96.3	Hemophilus influenzae [H. influenzae] as the cause of diseases classified elsewhere
B96.4	Proteus (mirabilis) (morganii) as the cause of diseases classified elsewhere
B96.5	Pseudomonas (aeruginosa) (mallei) (pseudomallei) as the cause of diseases classified elsewhere
B96.6	Bacteroides fragilis [B. fragilis] as the cause of diseases classified elsewhere
B96.7	Clostridium perfringens [C. perfringens] as the cause of diseases classified elsewhere
B96.89	Other specified bacterial agents as the cause of diseases classified elsewhere
H05.019	Orbital cellulitis
H05.021	osteomyelitis of right orbit
H05.022	osteomyelitis of left orbit
H05.023	osteomyelitis of bilateral orbits
H05.029	osteomyelitis of unspecified orbit
J02.0	Streptococcal throat/scarlet fever
J03.00	Acute streptococcal tonsillitis, unspecified
J13	Pneumococcal pneumonia (streptococcus pneumoniae pneumonia)
J14	Pneumonia due to HEMOPHILUS INFLUENZAE (H. INFLUENZAE)
J15.0	Pneumonia due to KLEBSIELLA PNEUMONIAE
J15.1	Pneumonia due to PSEUDOMONAS
J15.20	Pneumonia due to Staphylococcus unspecified
J15.211	Methicillin susceptible pneumonia due to staphylococcus aureus
J15.212	Methicillin resistant pneumonia due to staphylococcus aureus
J15.29	Other staphylococcus pneumonia
J15.3	Pneumonia due to Streptococcus Group B
J15.4	Pneumonia due to other Streptococcus
J15.5	Pneumonia due to Escherichia Coli (E. Coli)
J15.6	Pneumonia due to other Gram-negative bacteria
J15.7	Pneumonia due to mycoplasma pneumonia
J15.8	Pneumonia due to other specified bacteria
J15.9	Bacterial pneumonia unspecified
J16.0	Pneumonia due to chlamydia
J16.8	Pneumonia due to other specified organism
J18.1	Lobar pneumonia, unspecified organism
J20.0	acute bronchitis due to mycoplasma pneumoniae
J20.2	acute bronchitis due to streptococcus
J340	abscess, furuncle and carbuncle of nose
J36	Peritonsillar abscess
J38.7	Cellulitis and perichondritis of larynx
J39.1	Other abscess of pharynx
J85.0	Gangrene and necrosis of lung
J85.1	Abscess of lung with pneumonia
J85.2	Abscess of lung without pneumonia
J85.3	Abscess of mediastinum
J95.4	Postprocedural aspiration pneumonia
J95.851	Ventilator associated pneumonia
K12.2	Cellulitis and abscess of mouth
K68.11	Postprocedural retroperitoneal abscess
L01.00	Impetigo, unspecified
L01.03	Bullous impetigo
L02.02	furuncle of face
L02.03	carbuncle of face
L02.12	furuncle of neck
L02.13	carbuncle of neck
L02.221	furuncle of abdominal wall
L02.222	furuncle of back [any part, except buttock]
L02.223	furuncle of chest wall
L02.224	furuncle of groin
L02.225	furuncle of perineum
L02.226	furuncle of umbilicus

© 2021 Gracner et al. *JAMA Network Open*.

L02.229	furuncle of trunk, unspecified
L02.231	carbuncle of abdominal wall
L02.232	carbuncle of back [any part, except,buttock]
L02.233	carbuncle of chest wall
L02.234	carbuncle of groin of groin
L02.235	carbuncle of perineum of perineum
L02.236	carbuncle of umbilicus of umbilicus
L02.239	carbuncle of trunk, unspecified of trunk, unspecified
L02.32	furuncle of buttock
L02.33	carbuncle of buttock of buttock
L02.421	furuncle of right axilla
L02.422	furuncle of left axilla
L02.423	furuncle of right upper limb
L02.424	furuncle of left upper limb
L02.425	furuncle of right lower limb
L02.426	furuncle of left lower limb
L02.429	furuncle of limb, unspecified
L02.431	carbuncle of right axilla of right axilla
L02.432	carbuncle of left axilla of left axilla
L02.433	carbuncle of right upper limb of right upper limb
L02.434	carbuncle of left upper limb of left upper limb
L02.435	carbuncle of right lower limb of right lower limb
L02.436	carbuncle of left lower limb of left lower limb
L02.439	carbuncle of limb, unspecified of limb, unspecified
L02.521	furuncle right hand
L02.522	furuncle left hand
L02.529	furuncle unspecified hand
L02.531	carbuncle of right hand of right hand
L02.532	carbuncle of left hand of left hand
L02.539	carbuncle of unspecified hand of unspecified hand
L02.621	furuncle of right foot
L02.622	furuncle of left foot
L02.629	furuncle of unspecified foot
L02.631	carbuncle of right foot of right foot
L02.632	carbuncle of left foot of left foot
L02.639	carbuncle of unspecified foot of unspecified foot
L02.821	furuncle of head [any part, except face]
L02.828	furuncle of other sites
L02.831	carbuncle of head [any part, except,face] of head [any part, except,face]
L02.838	carbuncle of other sites of other sites
L02.92	furuncle, unspecified
L02.93	Carbuncle, unspecified
L03.011	cellulitis of right finger
L03.012	cellulitis of left finger
L03.019	Cellulitis of unspecified finger
L03.029	Acute lymphangitis of unspecified finger
L03.031	cellulitis of right toe
L03.032	cellulitis of left toe
L03.039	Cellulitis of unspecified toe
L03.049	Acute lymphangitis of unspecified toe
L031.13	cellulitis of right upper limb
L031.14	cellulitis of left upper limb
L031.15	cellulitis of right lower limb
L031.16	cellulitis of left lower limb
L03.119	Cellulitis of unspecified part of limb
L03.129	Acute lymphangitis of unspecified part of limb
L03.211	Cellulitis of face
L03.212	Acute lymphangitis of face
L03.213	Periorbital cellulitis
L03.221	Cellulitis of neck
L03.222	Acute lymphangitis of neck
L03.311	cellulitis of abdominal wall
L03.312	cellulitis of back [any part except buttock]
L03.313	cellulitis of chest wall
L03.314	cellulitis of groin
L03.315	cellulitis of perineum

© 2021 Gracner et al. *JAMA Network Open*.

L03.316	cellulitis of umbilicus
L03.317	Cellulitis of buttock
L03.319	Cellulitis of trunk, unspecified
L03.329	Acute lymphangitis of trunk, unspecified
L03.811	Cellulitis of head [any part, except face]
L03.818	Cellulitis of other sites
L03.891	Acute lymphangitis of head [any part, except face]
L03.898	Acute lymphangitis of other sites
L03.90	Cellulitis, unspecified
L03.91	Acute lymphangitis, unspecified
L08.0	Pyoderma
L08.81	Other pyoderma
L08.89	Other specified local infections of the skin and subcutaneous tissue
L08.9	Local infection of the skin and subcutaneous tissue, unspecified
M72.6	Necrotizing fasciitis
L88	Pyoderma gangrenosum
L98.0	Pyogenic granuloma
M00.00	Staphylococcal arthritis, unspecified joint
M00.019	Staphylococcal arthritis, unspecified shoulder
M00.029	Staphylococcal arthritis, unspecified elbow
M00.039	Staphylococcal arthritis, unspecified wrist
M00.049	Staphylococcal arthritis, unspecified wrist
M00.059	Staphylococcal arthritis, unspecified hip
M00.069	Staphylococcal arthritis, unspecified knee
M00.079	Staphylococcal arthritis, unspecified ankle and foot
M00.08	Staphylococcal arthritis, vertebrae
M00.09	Staphylococcal polyarthritis
M00.10	Pneumococcal arthritis, unspecified joint
M00.119	Pneumococcal arthritis, unspecified shoulder
M00.129	Pneumococcal arthritis, unspecified elbow
M00.139	Pneumococcal arthritis, unspecified wrist
M00.149	Pneumococcal arthritis, unspecified hand
M00.159	Pneumococcal arthritis, unspecified hip
M00.169	Pneumococcal arthritis, unspecified knee
M00.179	Pneumococcal arthritis, unspecified ankle and foot
M00.18	Pneumococcal arthritis, vertebrae
M00.19	Pneumococcal polyarthritis
M00.20	Other streptococcal arthritis, unspecified joint
M00.219	Other streptococcal arthritis, unspecified shoulder
M00.229	Other streptococcal arthritis, unspecified elbow
M00.239	Other streptococcal arthritis, unspecified wrist
M00.249	Other streptococcal arthritis, unspecified hand
M00.259	Other streptococcal arthritis, unspecified hip
M00.269	Other streptococcal arthritis, unspecified knee
M00.279	Other streptococcal arthritis, unspecified ankle and foot
M00.28	Other streptococcal arthritis, vertebrae
M00.29	Other streptococcal polyarthritis
M00.80	Arthritis due to other bacteria, unspecified joint
M00.819	Arthritis due to other bacteria, unspecified shoulder
M00.829	Arthritis due to other bacteria, unspecified elbow
M00.839	Arthritis due to other bacteria, unspecified wrist
M00.849	Arthritis due to other bacteria, unspecified hand
M00.859	Arthritis due to other bacteria, unspecified hip
M00.869	Arthritis due to other bacteria, unspecified knee
M00.879	Arthritis due to other bacteria, unspecified ankle and foot
M00.88	Arthritis due to other bacteria, vertebrae
M00.89	Polyarthritis due to other bacteria
M00.9	Pyogenic arthritis, unspecified
M46.20	osteomyelitis of vertebra, site unspecified
M46.21	osteomyelitis of vertebra, occipito-atlanto-axial region
M46.22	osteomyelitis of vertebra, cervical region
M46.23	osteomyelitis of vertebra, cervicothoracic region
M46.24	osteomyelitis of vertebra, thoracic region
M46.25	osteomyelitis of vertebra, thoracolumbar region
M46.26	osteomyelitis of vertebra, lumbar region
M46.27	osteomyelitis of vertebra, lumbosacral region

© 2021 Gracner et al. *JAMA Network Open*.

M46.28	osteomyelitis of vertebra, sacral and sacrococcygeal region
M60.009	Infective myositis, unspecified site
M86.00	acute hematogenous osteomyelitis, unspecified site
M86.011	acute hematogenous osteomyelitis, right shoulder
M86.012	acute hematogenous osteomyelitis, left shoulder
M86.019	acute hematogenous osteomyelitis, unspecified shoulder
M86.021	acute hematogenous osteomyelitis, right humerus
M86.022	acute hematogenous osteomyelitis, left humerus
M86.029	acute hematogenous osteomyelitis, unspecified humerus
M86.031	acute hematogenous osteomyelitis, right radius and ulna
M86.032	acute hematogenous osteomyelitis, left radius and ulna
M86.039	acute hematogenous osteomyelitis, unspecified radius and ulna
M86.041	acute hematogenous osteomyelitis, right hand
M86.042	acute hematogenous osteomyelitis, left hand
M86.049	acute hematogenous osteomyelitis, unspecified hand
M86.051	acute hematogenous osteomyelitis, right femur
M86.052	acute hematogenous osteomyelitis, left femur
M86.059	acute hematogenous osteomyelitis, unspecified femur
M86.061	acute hematogenous osteomyelitis, right tibia and fibula
M86.062	acute hematogenous osteomyelitis, left tibia and fibula
M86.069	acute hematogenous osteomyelitis, unspecified tibia and fibula
M86.071	acute hematogenous osteomyelitis, right ankle and foot
M86.072	acute hematogenous osteomyelitis, left ankle and foot
M86.079	acute hematogenous osteomyelitis, unspecified ankle and foot
M86.08	acute hematogenous osteomyelitis, other sites
M86.09	acute hematogenous osteomyelitis, multiple sites
M86.10	other acute osteomyelitis, unspecified site
M86.111	other acute osteomyelitis, right shoulder
M86.112	other acute osteomyelitis, left shoulder
M86.119	other acute osteomyelitis, unspecified shoulder
M86.121	other acute osteomyelitis, right humerus
M86.122	other acute osteomyelitis, left humerus
M86.129	other acute osteomyelitis, unspecified humerus
M86.131	other acute osteomyelitis, right radius and ulna
M86.132	other acute osteomyelitis, left radius and ulna
M86.139	other acute osteomyelitis, unspecified radius and ulna
M86.141	other acute osteomyelitis, right hand
M86.142	other acute osteomyelitis, left hand
M86.149	other acute osteomyelitis, unspecified hand
M86.151	other acute osteomyelitis, right femur
M86.152	other acute osteomyelitis, left femur
M86.159	other acute osteomyelitis, unspecified femur
M86.161	other acute osteomyelitis, right tibia and fibula
M86.162	other acute osteomyelitis, left tibia and fibula
M86.169	other acute osteomyelitis, unspecified tibia and fibula
M86.171	other acute osteomyelitis, right ankle and foot
M86.172	other acute osteomyelitis, left ankle and foot
M86.179	other acute osteomyelitis, unspecified ankle and foot
M86.18	other acute osteomyelitis, other site
M86.19	other acute osteomyelitis, multiple sites
M86.20	subacute osteomyelitis, unspecified site
M86.211	subacute osteomyelitis, right shoulder
M86.212	subacute osteomyelitis, left shoulder
M86.219	subacute osteomyelitis, unspecified shoulder
M86.221	subacute osteomyelitis, right humerus
M86.222	subacute osteomyelitis, left humerus
M86.229	subacute osteomyelitis, unspecified humerus
M86.231	subacute osteomyelitis, right radius and ulna
M86.232	subacute osteomyelitis, left radius and ulna
M86.239	subacute osteomyelitis, unspecified radius and ulna
M86.241	subacute osteomyelitis, right hand
M86.242	subacute osteomyelitis, left hand
M86.249	subacute osteomyelitis, unspecified hand
M86.251	subacute osteomyelitis, right femur
M86.252	subacute osteomyelitis, left femur
M86.259	subacute osteomyelitis, unspecified femur

© 2021 Gracner et al. *JAMA Network Open*.

M86.261	subacute osteomyelitis, right tibia and fibula
M86.262	subacute osteomyelitis, left tibia and fibula
M86.269	subacute osteomyelitis, unspecified tibia and fibula
M86.271	subacute osteomyelitis, right ankle and foot
M86.272	subacute osteomyelitis, left ankle and foot
M86.279	subacute osteomyelitis, unspecified ankle and foot
M86.28	subacute osteomyelitis, other site
M86.29	subacute osteomyelitis, multiple sites
M86.30	chronic multifocal osteomyelitis, unspecified site
M86.311	chronic multifocal osteomyelitis, right shoulder
M86.312	chronic multifocal osteomyelitis, left shoulder
M86.319	chronic multifocal osteomyelitis, unspecified shoulder
M86.321	chronic multifocal osteomyelitis, right humerus
M86.322	chronic multifocal osteomyelitis, left humerus
M86.329	chronic multifocal osteomyelitis, unspecified humerus
M86.331	chronic multifocal osteomyelitis, right radius and ulna
M86.332	chronic multifocal osteomyelitis, left radius and ulna
M86.339	chronic multifocal osteomyelitis, unspecified radius and ulna
M86.341	chronic multifocal osteomyelitis, right hand
M86.342	chronic multifocal osteomyelitis, left hand
M86.349	chronic multifocal osteomyelitis, unspecified hand
M86.351	chronic multifocal osteomyelitis, right femur
M86.352	chronic multifocal osteomyelitis, left femur
M86.359	chronic multifocal osteomyelitis, unspecified femur
M86.361	chronic multifocal osteomyelitis, right tibia and fibula
M86.362	chronic multifocal osteomyelitis, left tibia and fibula
M86.369	chronic multifocal osteomyelitis, unspecified tibia and fibula
M86.371	chronic multifocal osteomyelitis, right ankle and foot
M86.372	chronic multifocal osteomyelitis, left ankle and foot
M86.379	chronic multifocal osteomyelitis, unspecified ankle and foot
M86.38	chronic multifocal osteomyelitis, other site
M86.39	chronic multifocal osteomyelitis, multiple sites
M86.40	chronic osteomyelitis with draining sinus, unspecified site
M86.411	chronic osteomyelitis with draining sinus, right shoulder
M86.412	chronic osteomyelitis with draining sinus, left shoulder
M86.419	chronic osteomyelitis with draining sinus, unspecified shoulder
M86.421	chronic osteomyelitis with draining sinus, right humerus
M86.422	chronic osteomyelitis with draining sinus, left humerus
M86.429	chronic osteomyelitis with draining sinus, unspecified humerus
M86.431	chronic osteomyelitis with draining sinus, right radius and ulna
M86.432	chronic osteomyelitis with draining sinus, left radius and ulna
M86.439	chronic osteomyelitis with draining sinus, unspecified radius and ulna
M86.441	chronic osteomyelitis with draining sinus, right hand
M86.442	chronic osteomyelitis with draining sinus, left hand
M86.449	chronic osteomyelitis with draining sinus, unspecified hand
M86.451	chronic osteomyelitis with draining sinus, right femur
M86.452	chronic osteomyelitis with draining sinus, left femur
M86.459	chronic osteomyelitis with draining sinus, unspecified femur
M86.461	chronic osteomyelitis with draining sinus, right tibia and fibula
M86.462	chronic osteomyelitis with draining sinus, left tibia and fibula
M86.469	chronic osteomyelitis with draining sinus, unspecified tibia and fibula
M86.471	chronic osteomyelitis with draining sinus, right ankle and foot
M86.472	chronic osteomyelitis with draining sinus, left ankle and foot
M86.479	chronic osteomyelitis with draining sinus, unspecified ankle and foot
M86.48	chronic osteomyelitis with draining sinus, other site
M86.49	chronic osteomyelitis with draining sinus, multiple sites
M86.50	other chronic hematogenous osteomyelitis, unspecified site
M86.511	other chronic hematogenous osteomyelitis, right shoulder
M86.512	other chronic hematogenous osteomyelitis, left shoulder
M86.519	other chronic hematogenous osteomyelitis, unspecified shoulder
M86.521	other chronic hematogenous osteomyelitis, right humerus
M86.522	other chronic hematogenous osteomyelitis, left humerus
M86.529	other chronic hematogenous osteomyelitis, unspecified humerus
M86.531	other chronic hematogenous osteomyelitis, right radius and ulna
M86.532	other chronic hematogenous osteomyelitis, left radius and ulna
M86.539	other chronic hematogenous osteomyelitis, unspecified radius and ulna

© 2021 Gracner et al. *JAMA Network Open*.

M86.541	other chronic hematogenous osteomyelitis, right hand
M86.542	other chronic hematogenous osteomyelitis, left hand
M86.549	other chronic hematogenous osteomyelitis, unspecified hand
M86.551	other chronic hematogenous osteomyelitis, right femur
M86.552	other chronic hematogenous osteomyelitis, left femur
M86.559	other chronic hematogenous osteomyelitis, unspecified femur
M86.561	other chronic hematogenous osteomyelitis, right tibia and fibula
M86.562	other chronic hematogenous osteomyelitis, left tibia and fibula
M86.569	other chronic hematogenous osteomyelitis, unspecified tibia and fibula
M86.571	other chronic hematogenous osteomyelitis, right ankle and foot
M86.572	other chronic hematogenous osteomyelitis, left ankle and foot
M86.579	other chronic hematogenous osteomyelitis, unspecified ankle and foot
M86.58	other chronic hematogenous osteomyelitis, other site
M86.59	other chronic hematogenous osteomyelitis, multiple sites
M86.60	other chronic osteomyelitis, unspecified site
M86.611	other chronic osteomyelitis, right shoulder
M86.612	other chronic osteomyelitis, left shoulder
M86.619	other chronic osteomyelitis, unspecified shoulder
M86.621	other chronic osteomyelitis, right humerus
M86.622	other chronic osteomyelitis, left humerus
M86.629	other chronic osteomyelitis, unspecified humerus
M86.631	other chronic osteomyelitis, right radius and ulna
M86.632	other chronic osteomyelitis, left radius and ulna
M86.639	other chronic osteomyelitis, unspecified radius and ulna
M86.641	other chronic osteomyelitis, right hand
M86.642	other chronic osteomyelitis, left hand
M86.649	other chronic osteomyelitis, unspecified hand
M86.651	other chronic osteomyelitis, right thigh
M86.652	other chronic osteomyelitis, left thigh
M86.659	other chronic osteomyelitis, unspecified thigh
M86.661	other chronic osteomyelitis, right tibia and fibula
M86.662	other chronic osteomyelitis, left tibia and fibula
M86.669	other chronic osteomyelitis, unspecified tibia and fibula
M86.671	other chronic osteomyelitis, right ankle and foot
M86.672	other chronic osteomyelitis, left ankle and foot
M86.679	other chronic osteomyelitis, unspecified ankle and foot
M86.68	other chronic osteomyelitis, other site
M86.69	other chronic osteomyelitis, multiple sites
M86.8X0	other osteomyelitis, multiple sites
M86.8X1	other osteomyelitis, shoulder
M86.8X2	other osteomyelitis, upper arm
M86.8X3	other osteomyelitis, forearm
M86.8X4	other osteomyelitis, hand
M86.8X5	other osteomyelitis, thigh
M86.8X6	other osteomyelitis, lower leg
M86.8X7	other osteomyelitis, ankle and foot
M86.8X8	other osteomyelitis, other site
M86.8X9	other osteomyelitis, unspecified sites
M86.9	osteomyelitis, unspecified
N11.0	Kidney infection
N34.0	Urethral abscess
N39.0	Urinary tract infection site not specified
N41.0	Acute prostatitis
N71.0	Acute inflammatory disease of uterus
N72	Inflammatory disease of cervix uteri
N73.0	Acute parametritis and pelvic cellulitis
N99.511	Cystostomy infection
N99.89	Other postprocedural complications and disorders of genitourinary system
R65.10	SIRS (Systemic inflammatory response syndrome)
R65.20	Severe sepsis without septic shock
R65.21	Severe sepsis with septic shock
R78.81	Bacteremia
T80.211A	Bloodstream infection due to central venous catheter
T80.212A	Local infection due to central venous catheter
T80.219A	Other and unspecified infection due to central venous catheter
T81.4XXA	Infection following a procedure, initial encounter

© 2021 Gracner et al. *JAMA Network Open*.

T82.6XXA	Infection and inflammatory reaction due to cardiac valve prosthesis, initial encounter
T82.7XXA	Infection and inflammatory reaction due to other cardiac and vascular devices, implants and grafts, initial encounter
T83.51XA	Infection and inflammation due to indwelling urinary catheter
T83.590A	Infection and inflammatory reaction due to implanted urinary neurostimulation device, initial encounter
T83.591A	Infection and inflammatory reaction due to implanted urinary sphincter, initial encounter
T83.592A	Infection and inflammatory reaction due to indwelling ureteral stent, initial encounter
T83.593A	Infection and inflammatory reaction due to other urinary stents, initial encounter
T83.598A	Infection and inflammatory reaction due to other prosthetic device, implant and graft in urinary system, initial encounter
T83.61XA	Infection and inflammatory reaction due to implanted penile prosthesis, initial encounter
T83.62XA	Infection and inflammatory reaction due to implanted testicular prosthesis, initial encounter
T83.69XA	Infection and inflammatory reaction due to other prosthetic device, implant and graft in genital tract, initial encounter
T84.50XA	Infection and inflammatory reaction due to unspecified internal joint prosthesis, initial encounter
T84.60XA	Infection and inflammatory reaction due to internal fixation device of unspecified site, initial encounter
T84.7XXA	Infection and inflammatory reaction due to other internal orthopedic prosthetic devices, implants and grafts, initial encounter
T85.71XA	Infection and inflammatory reaction due to peritoneal dialysis catheter, initial encounter
T85.730A	Infection and inflammatory reaction due to ventricular intracranial (communicating) shunt, initial encounter
T85.731A	Infection and inflammatory reaction due to implanted electronic neurostimulator of brain, electrode (lead), initial encounter
T85.732A	Infection and inflammatory reaction due to implanted electronic neurostimulator of peripheral nerve, electrode (lead), initial encounter
T85.733A	Infection and inflammatory reaction due to implanted electronic neurostimulator of spinal cord, electrode (lead), initial encounter
T85.734A	Infection and inflammatory reaction due to implanted electronic neurostimulator, generator, initial encounter
T85.735A	Infection and inflammatory reaction due to cranial or spinal infusion catheter, initial encounter
T85.738A	Infection and inflammatory reaction due to other nervous system device, implant or graft, initial encounter
T85.79XA	Infection and inflammatory reaction due to other internal prosthetic devices, implants and grafts, initial encounter
A04.72	enterocolitis due to clostridium difficile, not specified as recurrent
Z16.10	resistance to unspecified beta lactam antibiotics
Z16.11	resistance to penicillins
Z16.12	extended spectrum beta lactamase (esbl) resistance
Z16.19	resistance to other specified beta lactam antibiotics
Z16.20	resistance to unspecified antibiotic
Z16.21	resistance to vancomycin
Z16.22	resistance to vancomycin related antibiotics
Z16.23	resistance to quinolones and fluoroquinolones
Z16.24	resistance to multiple antibiotics
Z16.29	resistance to other single specified antibiotic
Z16.30	resistance to unspecified antimicrobial drugs
Z16.31	resistance to antiparasitic drug(s)
Z16.341	resistance to single antimycobacterial drug
Z16.342	resistance to multiple antimycobacterial drugs
A08.8	Other specified intestinal infections
A09	Infectious gastroenteritis and colitis, unspecified
B99.9	Pneumonia in infectious diseases classified elsewhere
J00	Acute nasopharyngitis
J01.00	Acute maxillary sinusitis
J01.10	Acute frontal sinusitis
J01.20	Acute ethmoidal sinusitis
j01.30	Acute sphenoidal sinusitis
j01.40	Other acute sinusitis
j01.90	Acute sinusitis unspecified
j02.9	Acute pharyngitis
j03.90	Acute tonsillitis
j04.0	Acute laryngitis and tracheitis
j04.10	Acute tracheitis without obstruction
j04.11	Acute tracheitis with obstruction
j04.20	Acute laryngotracheitis without obstruction
J04.30	Supraglottitis unspecified without obstruction
J04.31	Supraglottitis unspecified with obstruction
j05.0	Acute obstructive laryngitis [croup]
j05.10	Acute epiglottitis without obstruction
j05.11	Acute epiglottitis with obstruction
J06.0	Acute laryngopharyngitis
J06.9	Acute upper respiratory infection, unspecified
J17	Pneumonia in diseases classified elsewhere
J18.0	Bronchopneumonia, unspecified organism
J18.2	Hypostatic pneumonia, unspecified organism

© 2021 Gracner et al. *JAMA Network Open*.

J18.8	Pneumonia organism unspecified
J18.9	Pneumonia, unspecified organism
J20.9	Acute bronchitis
J22	unspecified acute lower respiratory infection
J44.0	Chronic obstructive pulmonary disease with acute lower respiratory infection
L04.9	Acute lymphadenitis
T80.22XA	Acute infection following transfusion, infusion, or injection of blood and blood products, initial encounter
T80.29XA	Infection following other infusion, transfusion and therapeutic injection, initial encounter
T83.511A	infection and inflammatory reaction due to indwelling urethral catheter, initial encounter
T83.518A	infection and inflammatory reaction due to other urinary catheter, initial encounter
T88.0XXA	Infection following immunization, initial encounter

© 2021 Gracner et al. *JAMA Network Open*.

eTable 9: ICD-9 codes identifying sepsis-related complications.

ICD-9 code	Description
036.2	Meningococcal septicemia
038.0	Streptococcal septicemia
038.10	Staphylococcal septicemia NOS
038.11	MSSA septicemia
038.12	MRSA septicemia
038.19	Other staphylococcal septicemia
038.2	Pneumococcal septicemia
038.3	Anaerobic septicemia
038.40	Gram-negative septicemia NOS
038.41	H. influenzae septicemia
038.42	E. coli septicemia
038.43	Pseudomonas septicemia
038.44	Serratia septicemia
038.49	Gram-neg septicemia NEC
038.8	Septicemia NEC
038.9	Septicemia NOS
785.52	Septic shock
790.7	Bacteremia
995.90	SIRS (Systemic inflammatory response syndrome)
995.91	Sepsis
995.92	Severe sepsis
020.0	Plague
020.1	Plague
020.2	Septicemic plague
020.3	Plague
020.4	Plague
020.5	Plague
020.8	Plague
020.9	Plague
021	Tularemia
022	Anthrax
023	Brucellosis
024	Glanders
025	Meliodiosis
026	Rat-bite fever
027	Other bacterial zoonoses
032	Pharyngeal diphtheria
033	Whooping cough
034	Streptococcal throat/scarlet fever
037	Tetanus
040	Other bacterial diseases
091	Early syphilis, symptomatic
092	Early syphilis, latent
095	Other forms of late syphilis, with symptoms

© 2021 Gracner et al. *JAMA Network Open*.

eTable 10: ICD-10 codes identifying sepsis-related complications.

ICD-10 code	Description
A20.0	Plague
A20.1	Plague
A20.2	Plague
A20.7	Septicemic plague
A20.8	Plague
A20.9	Plague
A21.0	Tularemia
A21.1	Tularemia
A21.2	Tularemia
A21.3	Tularemia
A21.7	Tularemia
A21.8	Tularemia
A21.9	Tularemia
A22.0	Anthrax
A22.1	Anthrax
A22.2	Anthrax
A22.7	Anthrax
A22.8	Anthrax
A22.9	Anthrax
A23.0	Brucellosis
A23.1	Brucellosis
A23.2	Brucellosis
A23.3	Brucellosis
A23.8	Brucellosis
A23.9	Brucellosis
A24.0	Glanders
A24.3	Melioidosis
A24.9	Melioidosis
A25.0	Spirillosis
A27.0	Leptospirosis
A32.7	Listerial sepsis
A35	Tetanus
A36.0	Diphteria
A37.00	Whooping cough
A39.4	Meningococcal sepsis
A40.0	sepsis due to streptococcus, group a
A40.1	sepsis due to streptococcus, group b
A40.8	other streptococcal sepsis
A41.81	sepsis due to enterococcus
A40.3	sepsis due to streptococcus pneumoniae
A40.9	Streptococcal sepsis, unspecified
A41.01	MSSA sepsis
A41.02	MRSA sepsis
A41.1	Other staphylococcal sepsis
A41.2	Staphylococcal sepsis (unspecified)
A41.3	Sepsis due to hemophilus influenzae
A41.4	Sepsis due to anaerobes
A41.50	Gram-negative sepsis, unspecified
A41.51	Sepsis due to e. coli
A41.52	Sepsis due to pseudomonas
A41.53	Sepsis due to serratia
A41.59	Other Gram-negative sepsis
A41.89	Other specified sepsis
A41.9	Unspecified sepsis
A48.0	Gas gangrene
A51.0	Primary genital syphilis
A51.5	Early syphilis, latent
A52.71	Late syphilitic oculopathy
J02.0	Streptococcal pharyngitis
J03.00	Acute streptococcal tonsillitis, unspecified
N11.0	Kidney infection
N34.0	Urethral abscess
N41.0	Acute prostatitis
N71.0	Acute inflammatory disease of uterus

© 2021 Gracner et al. *JAMA Network Open*.

N72	Inflammatory disease of cervix uteri
R65.10	SIRS (Systemic inflammatory response syndrome)
R65.20	Severe sepsis without septic shock
R65.21	Severe sepsis with septic shock
R78.81	Bacteremia
L04.9	Acute lymphadenitis

eReferences

1. Hospital Transfer Rates Among US Nursing Home Residents With Advanced Illness Before and After Initiatives to Reduce Hospitalizations | Cardiology | JAMA Internal Medicine | JAMA Network. Accessed December 21, 2020. https://jamanetwork.com/journals/jamainternalmedicine/fullarticle/2757790

2. Thomas KS, Dosa D, Wysocki A, Mor V. The Minimum Data Set 3.0 Cognitive Function Scale. *Med Care*. 2017;55(9):e68-e72. doi:10.1097/MLR.0000000000000334

3. Saliba D, Buchanan J, Edelen MO, et al. MDS 3.0: brief interview for mental status. *J Am Med Dir Assoc*. 2012;13(7):611-617. doi:10.1016/j.jamda.2012.06.004

4. Chodosh J, Edelen MO, Buchanan JL, et al. Nursing home assessment of cognitive impairment: development and testing of a brief instrument of mental status. *J Am Geriatr Soc*. 2008;56(11):2069-2075. doi:10.1111/j.1532-5415.2008.01944.x

5. Morris JN, Fries BE, Mehr DR, et al. MDS Cognitive Performance Scale. *J Gerontol*. 1994;49(4):M174-182. doi:10.1093/geronj/49.4.m174

6. Inouye SK, Van Dyck CH, Alessi CA, et al. Clarifying confusion: the confusion assessment method: a new method for detection of delirium. *Ann Intern Med.* 1990;113:941–948.

7. Wong CL, Holroyd-Leduc J, Simel DL, et al. Does this patient have delirium?: value of bedside instruments. *JAMA.* 2010;304:779–786.

8. Wei LA, Fearing MA, Sternberg EJ, et al. The Confusion Assessment Method: a systematic review of current usage. *J Am Geriatr Soc.* 2008;56:823–830.

9. Kosar CM, Thomas KS, Inouye SK, Mor V. Delirium during postacute nursing home admission and risk for adverse outcomes. Journal of the American Geriatrics Society. 2017 Jul;65(7):1470-5.

10. Cohen B, Liu J, Cohen AR, Larson E. Association between healthcare-associated infection and exposure to hospital roommates and previous bed occupants with the same organism. Infection control and hospital epidemiology. 2018 May;39(5):541.

© 2021 Gracner et al. *JAMA Network Open*.

11. Harrison JM, Agarwal M, Stone PW, Gracner T, Sorbero M, Dick AW. Does Integration of Palliative Care and Infection Management Reduce Hospital Transfers among Nursing Home Residents?. Journal of Palliative Medicine. 2021 Feb 19

© 2021 Gracner et al. *JAMA Network Open*.
